# Nano-Engineered Interfaces in Dual-Layer Electrodes for Protonic Ceramic Cells with Enhanced Stability and Kinetics

**DOI:** 10.1021/acsnano.5c15759

**Published:** 2025-12-08

**Authors:** Yuqi Geng, Shuanglin Zheng, Saroj Karki, Alejandro Serrano, Yijie Jiang, Bello Idris, Anshu Kumari, Dongyang Cao, Hanping Ding

**Affiliations:** School of Aerospace and Mechanical Engineering, 6187 University of Oklahoma, Norman, Oklahoma 73019, United States

**Keywords:** protonic ceramic cells, nanoengineered interfaces, interfacial bonding and charge transfer, dynamic operation and stability, faradaic efficiency

## Abstract

Enhancing interfacial stability and charge transfer in protonic ceramic cells (PCCs) remains a critical challenge, as structural degradation and interfacial resistance often compromise durability and efficiency. Here, we report a nanoengineered dual-layer oxygen electrode architecture designed to address these limitations by introducing a fine-grained nanoparticle interfacial contact layer beneath a porous catalytic backbone. The nanoscale powders, through enhanced sintering activity, densify into a robust interfacial layer that promotes strong chemical bonding, uniform adhesion, and continuous ionic/electronic pathways with the BCZYYb electrolyte. This hierarchical architecture mitigates delamination, redistributes mechanical stress, and establishes efficient charge and mass transport channels without relying on corrosive surface treatments. Electrochemical evaluation demonstrates that the dual-layer design markedly reduces interfacial polarization resistance and accelerates electrode kinetics. Compared to the single-layer counterpart, the architecture achieves a peel strength of 44.53 N/cm^2^, a 40% improvement in peak power density (0.96 W cm^–2^ at 600 °C), and a 130% enhancement in electrolysis current density (4.78 A cm^–2^ at 1.57 V). Faradaic efficiency remains as high as 88% under high steam concentrations, underscoring minimal charge loss during practical operation. Notably, the electrode retains stability across 450–600 °C and under transient voltage cycling, with impedance spectra confirming suppressed interfacial resistance growth over prolonged use. These results highlight nanoscale interface engineering as a powerful route to enhance both mechanical robustness and electrochemical kinetics in PCCs. The demonstrated scalability and durability of this architecture provide a versatile platform for advancing solid-state electrochemical systems, including reversible fuel cells and high-efficiency hydrogen production technologies.

## Graphical abstract

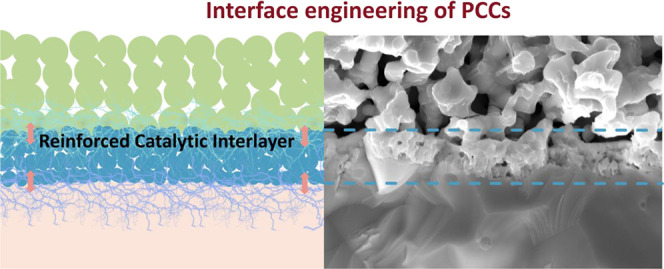

## 1 Introduction

The global shift toward decarbonization and renewable energy systems underscores the urgent need for highly efficient, sustainable energy conversion and storage technologies. The intermittent nature of solar and wind power generation necessitates reliable energy carriers—among which hydrogen stands out for its high energy density, carbon-free usage, and broad sectoral applicability.^1–10^ To effectively harness hydrogen as an energy carrier within renewable energy systems, efficient and reversible electrochemical devices are essential. Among the various technologies under development, protonic ceramic cells (PCCs) have emerged as a particularly promising platform due to their unique operating characteristics and versatility.^11–13^

One of the most compelling advantages of PCCs is their ability to function reversibly—operating as both fuel cells and electrolysis cells, which makes them exceptionally well-suited for storing intermittent renewable energy.^14–17^ In electrolysis mode, excess electricity from solar or wind sources can be used to produce hydrogen from water vapor with high efficiency. This hydrogen can then be stored and later converted back into electricity via the same cell in fuel-cell mode when renewable generation is low or demand spikes. This dual functionality allows PCCs to serve as flexible, dispatchable energy storage systems that can buffer fluctuations in renewable supply and stabilize the electrical grid. Their intermediate operating temperature range (400–700 °C) offers a practical balance between fast electrode kinetics and long-term material stability, making them particularly advantageous for dynamic cycling applications compared to high-temperature SOECs or low-temperature fuel cell systems.^18–24^ Moreover, the use of solid-state ceramic components enables robust operation under variable load conditions and with diverse fuel inputs, further enhancing their utility as a backbone technology for integrated, carbon-neutral energy infrastructures.

Despite these promising advantages and considerable progress over the past decade in the development of electrolyte materials, electrode architectures, and system configurations, PCCs still face several formidable challenges that must be addressed to enable widespread commercial deployment.^25–31^ Among these, one of the most critical and persistent bottlenecks lies in the engineering and stability of interfaces, particularly at the electrolyte/electrode boundaries. These interfaces play a pivotal role in determining the overall performance, efficiency, and durability of the cell, as they govern the charge transfer kinetics, catalytic activity, gas diffusion, and mechanical integrity of the device. One major interfacial challenge is the formation of resistive secondary phases at the electrolyte/electrode junctions, often due to undesirable reactions between the electrolyte and electrode materials during high-temperature sintering. These reaction products can impede ionic or electronic transport, introduce porosity or delamination, and increase polarization resistance.^32–36^ Additionally, poor adhesion at the interface can lead to mechanical degradation such as cracking, delamination, or grain boundary separation under thermal or redox cycling, particularly given the mismatched thermal expansion coefficients between different materials.

To enable safer, more controllable, and scalable interfacial bonding in PCCs, a variety of engineering strategies have been explored. Functionally graded interlayers and reactive sintered layers have been employed to reduce thermal mismatch and improve adhesion, though they often require complex fabrication processes and risk undesirable interfacial phase formation. Thin-film deposition techniques such as atomic layer deposition and pulsed laser deposition offer excellent control over interfacial chemistry but are constrained by high cost and limited scalability.^37^ Alternatively, surface reconstruction or chemical modification methods are simpler and more accessible but frequently suffer from poor reproducibility and limited control over surface morphology and composition. Recent studies have shown that brief nitric acid etching of well-annealed electrolyte surface can substantially enhance interfacial bonding, restore proton conductivity, and enable high performance in both fuel cell and electrolysis modes.^38^ However, this approach involves complex surface preparation and poses challenges in achieving precise control over etching depth and surface uniformity, which can affect reproducibility and long-term reliability. Collectively, these limitations highlight the need for an interfacial design strategy that offers precise control, structural reliability, and scalability, while avoiding complex or hard-to-reproduce processing steps.

Motivated by the need for a more controllable and environmentally friendly approach to interface engineering, we developed nanomodified dual layers electrode architecture designed to enhance interfacial bonding without the use of corrosive chemical treatments. More recently, the incorporation of nanosized fine particles has emerged as a highly promising approach. Owing to their significantly higher surface energy density, nano particles exhibit much stronger interfacial bonding with electrolytes, enhanced sintering activity, and improved adhesion reliability. These features not only promote denser and more stable electrode/electrolyte interfaces but also enable superior electrochemical performance in both fuel cell and electrolysis modes. Collectively, these findings highlight that nanoengineered particles provide a scalable and structurally reliable pathway for interfacial design, offering clear advantages over conventional processing strategies. The PrNi_0.7_Co_0.3_O_3−δ_(PNC) composition was selected based on prior work by Tang et al.^20^ The Ni-rich composition enhances hydration, lowers proton migration barriers, and enables measurable H_2_ permeation, effectively enlarging the reaction zone. In our design, a thin, nanostructured oxygen electrode PNC layer serves as a contact layer to promote strong bonding with the electrolyte, while a thicker, porous PNC top layer ensures sufficient catalytic activity and gas diffusion.^39^ This nanobridging architecture effectively enhances electrode–electrolyte adhesion, suppresses interfacial reactions, and facilitates charge transfer, all while maintaining a fabrication process that is scalable, reproducible, and environmentally benign. In this study, we systematically investigate the interfacial bonding between the electrolyte and the PNC oxygen electrode. By comparing the mechanical, structural, and electrochemical characteristics of single-layer and dual-layer electrode configurations, we demonstrate that the dual-layer design achieves a peel strength of 44.53 N/cm^2^, significantly improving adhesion. Electrochemically, the dual-layer PCC reaches a peak power density of 0.96 W cm^–2^ in fuel cell mode and sustains a current density of 1.72 A cm^–2^ at 1.3 V under electrolysis mode at 600 °C. We compare previously reported dual-layer or graded electrode structures in protonic ceramic cells to elucidate common design principles and associated performance^40–44^ (Table S1, Supporting Information). The cell also exhibits excellent durability, maintaining a Faradaic efficiency of up to 87.4% under dynamic, high-steam conditions and stable performance during stepwise galvanostatic operation. These results highlight the effectiveness of the dual-layer electrode in improving both performance and interfacial stability compared to the single-layer counterpart.

## 2 Results and Discussion

### 2.1 Redesign a Nanobridging Architecture Oxygen Electrode for Improving Interfacial Bonding

The enhanced sintering ability of fine particles originates from their high surface curvature. According to the Gibbs–Thomson relation, the chemical potential of atoms at a curved surface is expressed as μ = μ_0_ + γΩκ, κ = $\frac{2}{R}$ , where μ_0_ is the chemical potential of a flat surface, γ (J·m^–2^) represents the free energy required to create a unit area of new surface, Ω is the atomic volume, κ is the mean curvature, and *R* is the particle radius. Thus, the chemical potential difference scales as Δμ ≈ $\frac{2\gamma \Omega }{R}$ , Smaller particles with smaller *R* have larger chemical potential gradients, thereby providing a stronger thermodynamic driving force for sintering.^45,46^ In the two-sphere model, the growth of the neck radius *x* relative to the particle radius *R* is given by^47^

1 $$\frac{x}{R}\propto {\left(\frac{D\gamma \Omega }{\kappa T}\cdot \frac{t}{R^{n}}\right)}^{m}$$

where *D* is the diffusion coefficient, κ*T* the thermal energy, and *t* the sintering time. The kinetic exponents vary with the diffusion mechanism: surface diffusion: *n* = 4, *m* = 1/7; grain boundary diffusion: *n* = 4, *m* = 1/6; volume diffusion: *n* = 3, *m* = 1/5. Thus, smaller *R* accelerates normalized neck growth (*x*/*R*).

As illustrated in Figure 1, conventional microsized PNC particles provide limited interfacial contact with the BCZYYb electrolyte, leaving voids and weak bonding regions that restrict the number of active reaction sites and hinder efficient charge transfer. According to eq 1, we preliminarily propose incorporating nanosized PNC particles, synthesized via top-down mechanical methods. Top-down mechanical synthesis refers to mechanically driven processing of bulk precursors (e.g., high-energy ball milling) that reduces particle size and can induce solid state mechanochemical reactions. In this work “top-down” simply denotes the layer ordering, a micron-scale top layer over a nanoparticle-sintered bottom layer. Reducing the primary particle size from the micrometer to the nanometer regime significantly increases the surface-to-volume (*S*/*V*) ratio, enhancing the density of under-coordinated surface atoms and the number of electrochemically active bonding sites at the electrode–electrolyte junction. This promotes superior wetting and adhesion, increases the number and quality of real contact microjunctions, and diminishes interfacial voids, allowing a larger fraction of current to flow through well-bonded pathways.^48–50^ Due to their much higher surface-to-volume ratio and enhanced surface reactivity, nanoscale particles are expected to provide a denser distribution of active bonding sites, promote intimate adhesion, and accelerate sintering activity during cosintering. In the dual-layer architecture, a nanofunctionalized fine-grained interfacial layer promotes stronger interfacial bonding and continuous ionic/electronic pathways, whereas the coarse-particle backbone preserves microporosity for gas transport and provides bulk structural support.^45–48^ The conceptual design highlights the potential of nanoenabled dual-layer electrodes to overcome interfacial limitations inherent to micrograined structures, thereby offering a promising direction for achieving improved electrochemical integration and long-term stability. This separation allows simultaneous minimization of interfacial polarization and maximization of electrode kinetics, achieving performance improvements that cannot be fully realized with a single-layer design.

**Figure 1:**
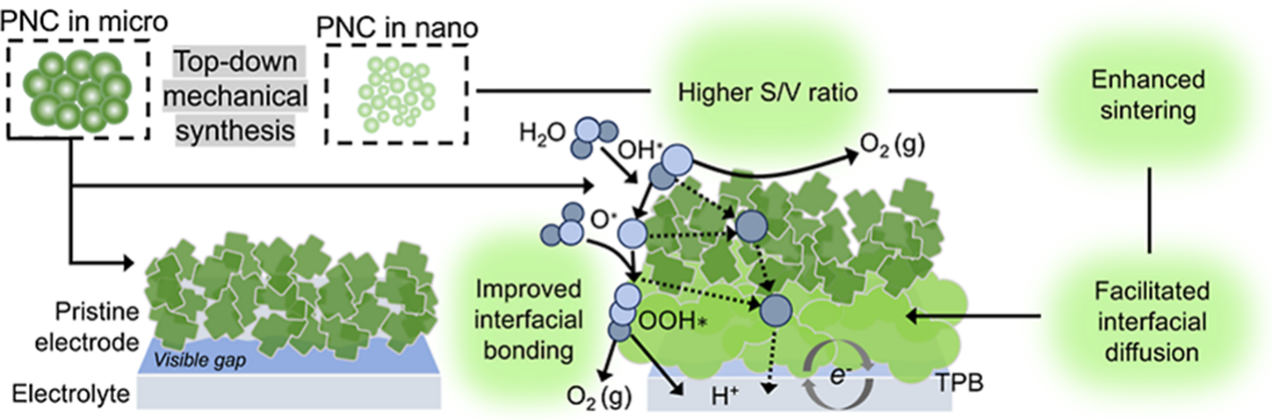
Nanoengineered dual-layer architecture bridging PNC electrodes and BCZYYb electrolyte. Schematic of the effects of finer PNC powder on electrode behavior. Reduced particle size increases surface to volume (*S*/*V*) ratio and interfacial bonding, leading to enhanced sintering, stronger coarsening, and improved oxygen electrocatalysis.

Figure 2 illustrates the design rationale, fabrication strategy, and cross-sectional microstructure of full cells incorporating a dual-layer oxygen electrode based on the PNC electrode. The cell fabrication process, as schematically shown in Figure 2a, begins with the preparation of a robust half-cell, consisting of a porous anode substrate (NiO + BCZYYb + corn starch), an anode functional layer (AFL, NiO + BCZYYb), and a dense electrolyte (BCZYYb) layer. These components are cosintered to form a structurally integrated base. The electrode adopts dual-layer architecture comprising a bottom layer of PNC nanoparticles and a top layer of pristine, coarse PNC particles. The electrode layers were applied onto the electrolyte surface using a brush-coating technique. We brushed the first nanoparticle PNC electrode, which was consequently cut to a precise area of 0.178 cm^2^ as the nano bridging electrode layer. After it was dry in the oven for 20 min, we painted the second pristine layer. Following this, the calcination process is carried out at 1050 °C for 2 h. This configuration is intentionally designed to fulfill two distinct but complementary roles: the nanoparticle layer ensures a highly conformal interface with the electrolyte, promoting strong interfacial bonding and facilitating oxygen ion transport, while the coarse particle layer provides high porosity and enhanced gas diffusion capabilities, which are crucial for sustaining the oxygen reduction reaction (ORR) at high rates.

**Figure 2:**
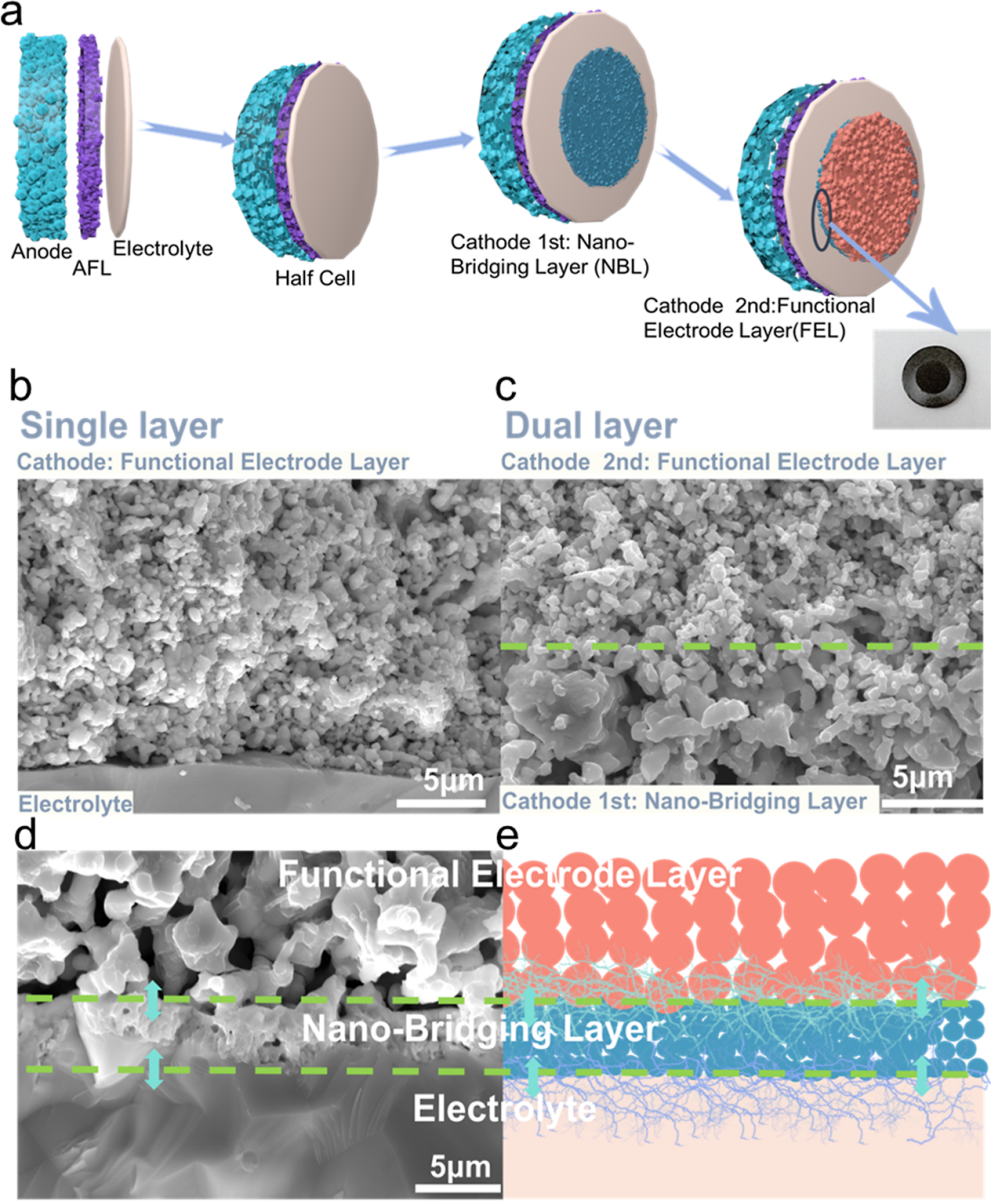
Formation process of the nanoparticle-engineered dual-layer electrode (PCC) with a nearly dense nanoscale bridging layer at the electrolyte interface, enabling enhanced interfacial durability and charge transfer. (a) Schematic illustration of the nanoparticle-driven formation of the dual-layer electrode architecture. (b,c) Cross-sectional comparison of interfacial morphologies in conventional single-layer and nanoparticle-assembled dual-layer electrodes. (d) Schematic illustration of the nanoscale bridging interlayer that ensures an intact and mechanically robust interface.

The interfacial characteristics between the dual-layer structured electrode and the underlying electrolyte are illustrated through cross-sectional scanning electron microscopy (SEM) images. Figure 2b shows a cross-section of a single-layer cathode made entirely of pristine PNC particles. The microstructure reveals a loosely packed arrangement of particles with significant interparticle voids and poor adhesion at the cathode-electrolyte interface. This weak interfacial bonding is primarily due to the large size and irregular morphology of the pristine particles, which prevent conformal contact with the electrolyte surface. These microstructural shortcomings lead to the formation of interfacial gaps and discontinuities that compromise both mechanical stability and electrochemical performance. From an electrochemical standpoint, the voids reduce the density of triple-phase boundaries (TPBs), which are crucial for efficient electrochemical reactions. Additionally, the poor interfacial contact increases polarization resistance at the electrode–electrolyte junction, hindering charge transfer process.^39,51,52^ As a result, these factors contribute to lower cell efficiency and reduced long-term operational stability.

In contrast, Figure 2c displays the microstructure of a nanobridging dual layer electrodes configuration, in which a nanoparticle layer is deposited directly onto the electrolyte, followed by the pristine particle layer on top. The interface between the nano bridging layer and the electrolyte is notably more compact and uniform, with fewer voids and stronger particle-to-particle contact. The PNC nanoparticles possess higher surface area and sinter more readily at moderate temperatures, which allows them to form a dense and adherent interfacial region. This structural modification not only enhances the electrochemical connectivity but also acts as a mechanical buffer layer, mitigating thermal mismatch and stress accumulation during repeated thermal cycling. The upper coarse layer, meanwhile, retains a porous and open morphology that facilitates gas transport and maximizes the electrochemically active surface area.

Further investigation of the interfacial region between the dual electrode layers and the electrolyte is provided by a high-magnification SEM image in Figure 2d, which reveals the formation of a bridging structure. This structure consists of PNC nanoparticles that infiltrate and partially bridge the interface between the electrolyte and the top pristine particle layer. Porosity analysis yields an interfacial porosity of 4.6%. This is very low value, which indicates that the layer is well densified and forms a continuous bonding layer with minimal unbonded voids (Figures S3, S4, Supporting Information). The bridging layer serves two key functions: it acts as an active layer with a high density of TPBs to enhance reaction activity, and it serves as both a mechanical and ionic bridge, integrating the coarse and fine layers. Such an interfacial configuration is vital for improving the long-term structural cohesion of the electrode and maintaining low interfacial resistance during operation. Figure 2e illustrates the dual-layer electrode with a nano bridging structure, dividing the electrode into three distinct functional regions: (1) a gas diffusion layer at the top, made up of loosely packed pristine PNC particles that allow for oxygen permeation; (2) an intermediate bridging and active layer formed by fine PNC particles, which offers a high TPB density and strong electrochemical activity; and (3) a tightly bonded bottom layer in contact with the electrolyte, ensuring efficient oxygen ion conduction into the electrolyte phase. This hierarchical, functionally graded design effectively separates the roles of gas diffusion and ionic conduction, while also alleviating stress concentrations and preventing microstructural degradation at the electrode/electrolyte interface. To assess elemental distribution across the PNC/BCZYYb interface, high-resolution SEM and EDS line scans were conducted (Figures S1, S2, S5–S17, Supporting Information). The interface shows continuous morphology without delamination, suggesting good adhesion. Subtle contrast variations imply possible interdiffusion during high-temperature processing, potentially forming a transition zone. Analysis of EDS profiles helps determine whether key elements (e.g., Pr, Co, Ni, Y, Zr, Ba, Ce, Yb) diffuse across the interface or if a chemically sharp boundary is maintained as an important factor for electrochemical performance and durability (Tables S2–S12, Supporting Information).

### 2.2 Enhanced Interfacial Bonding Strength and Its Effect on Oxygen Electrode Activity in Symmetric Cells

To investigate the performance differentials arising from electrode architecture, a comparative study was conducted between single- and dual-layer PNC electrodes interfaced with dense BCZYYb electrolyte. The analysis encompassed the examinations of interfacial mechanical adhesion, surface morphology, and electrochemical behavior in symmetrical cell under representative operating conditions.

Surface morphological characteristics, obtained by atomic force microscopy, further support these findings. While the electrolyte surface remains relatively smooth (*R*_q_ ≈ 0.11 μm, Figure 3a), the first layer (fine particles layer) of the dual-layer cathode exhibits substantially greater roughness (*R*_q_ ≈ 0.69 μm, Figure 3b), which not only facilitates mechanical interlock but also potentially amplifies the density of electrochemically active TPBs. The finer-grained cathode layer exhibits greater roughness compared to the electrolyte, making it easier to bond with the pristine cathode layer. Tensile tests also show that its adhesion to the electrolyte is stronger than that of the single layer. Therefore, the fine-grained nanolayer serves as a bridging interface. Such textural enhancement is considered beneficial for both interfacial stability and catalytic efficacy.^25,37,38^

**Figure 3:**
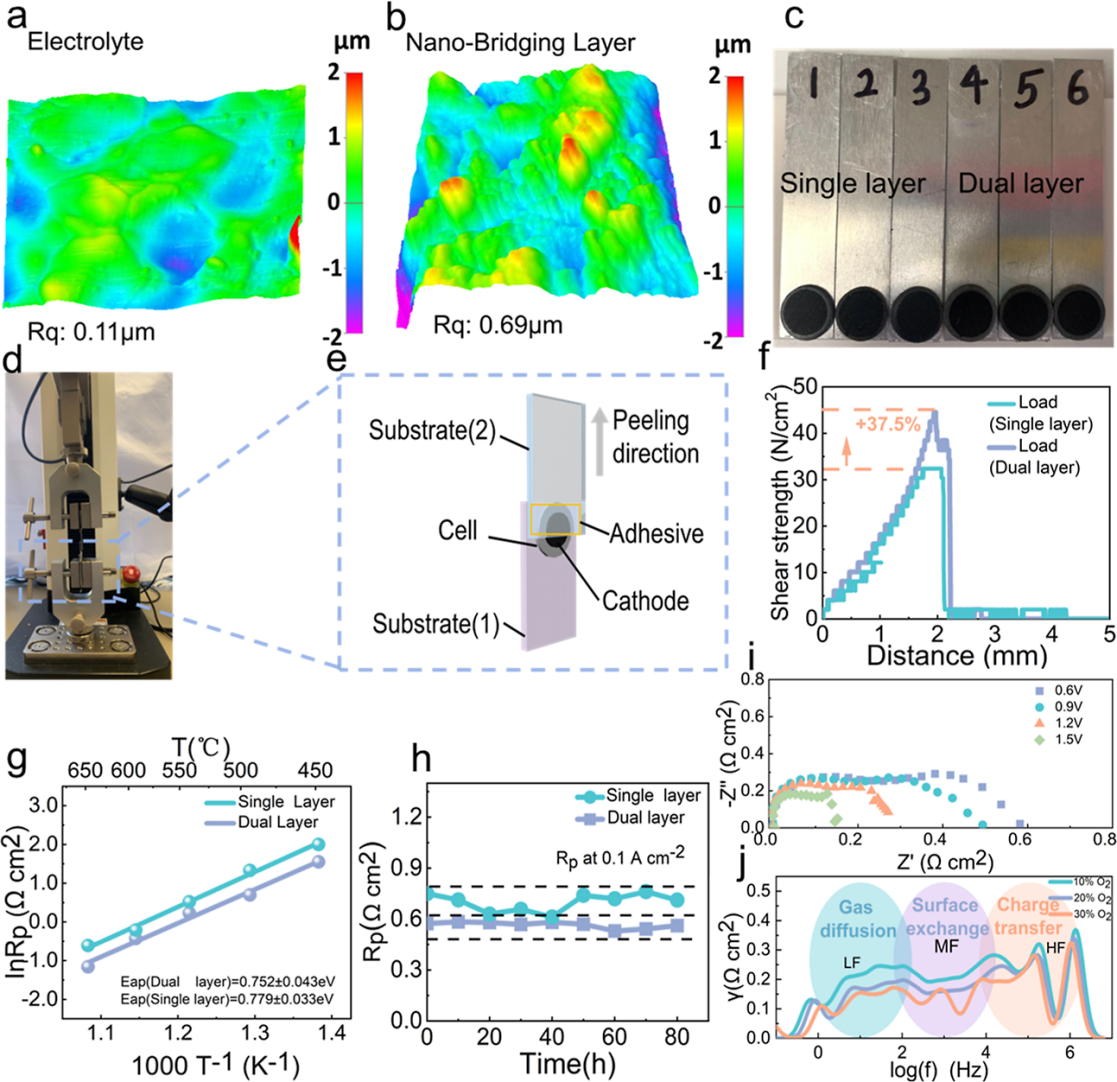
Mechanical and electrochemical analysis of single-layer and nanoparticle-engineered dual electrodes. (a) AFM image of the BCZYYb electrolyte surface. (b) AFM image of the PNC nanoparticles electrode surface. (c) Representative substrate samples. (d) Tensile testing apparatus. (e) Schematic of the interfacial peeling strength measurement procedure. (f) Shear strength measurement profiles for single and dual layer PNC electrodes. (g) Arrhenius plots of polarization resistance for single- and dual-layer electrodes from symmetric cells under open-circuit conditions in wet oxygen (∼3% H_2_O, O_2_:30 sccm). (h) Long-term stability of polarization resistance in 600 °C, ∼3% H_2_O, O_2_:30 sccm, confirming improved durability for the nanobridged dual-layer electrode. (i) EIS spectra of dual-layer PNC electrodes under varying applied voltages, showing voltage-dependent impedance characteristics. (j) DRT analysis at different oxygen partial pressures, illustrating oxygen-dependent electrochemical kinetics.

Interfacial adhesion strength was measured via a tensile-peeling test, as experimentally realized through the setup shown in Figure 3d and schematically illustrated in Figure 3e,c. This adhesion peeling test was conducted following the same principles as the ASTM standard (ASTM D1002-05). First, a stainless steel or aluminum sheet is selected as Substrate 1. The test specimen (e.g., a cell) is firmly bonded to one end of Substrate 1 using super glue, and the assembly is left to cure at room temperature for at least 12 h to ensure complete adhesion. After curing, double-sided tape is applied evenly to the other end of Substrate 2. Carefully aligning it with the specimen on Substrate 1, the two substrates are pressed together. Finally, uniform pressure applied by fingers or a roller helps form a strong, bubble-free bond. We performed multiple independent peel tests for every sample (*n* = 3) and presented error bars in (Figures S18, Supporting Information). From the force–displacement curves, it can be clearly observed that the single-layer cathode exhibits the peak shear strength of 32.38 N/cm^2^, while the dual-layer cathode achieves a notably higher value of 44.53 N/cm^2^ (Figure 3f). This corresponds to an approximate 37.5% increase in peak peeling force for the dual-layer configuration, indicating a pronounced enhancement in interfacial adhesion. This enhancement is attributed to improved mechanical interlocking and anchoring facilitated by the textured, fine-grained interfacial layer, which increases the effective contact area and promotes more uniform stress distribution across the electrode–electrolyte interface. The refined microstructure may also reduce local stress concentrations and inhibit crack initiation or propagation under mechanical loading.

Electrochemical impedance spectroscopy (EIS), conducted in symmetrical cell configuration under humidified oxygen (∼3% H_2_O), revealed clear architectural dependencies. As shown in Figure 3g, Arrhenius plots of polarization resistance (*R*_p_) exhibit lower values for dual-layer electrodes across the entire temperature range, with a corresponding decrease in apparent activation energy (*E*_a_ = 0.752 ± 0.043 eV for dual-layer vs 0.779 ± 0.033 eV for single-layer electrodes). Single-layer and dual-layer samples, the impedance spectra are fit using the same equivalent circuit: a series resistance (*R*_1_) in series with two parallel *R*-CPE (constant phase element) branches (*R*_2_∥CPE1 and *R*_3_∥CPE2). (Figure S19, Table S13, Supporting Information) This reduction indicates enhanced reaction kinetics, likely attributed to improved ionic percolation pathways facilitated by the interfacial electrode layer. Over an extended operational period (Figure 3h), the dual-layer electrode retained electrochemical stability more effectively, maintaining a consistent *R*_p_ for over 80 h, indicating of their robustness against degradation mechanisms such as delamination or microstructural coarsening.

To probe dynamic kinetic behavior under applied bias, the electrode was performed across a range of voltages (0.6 V, 0.9 V, 1.2 V, 1.5 V), respectively. The spectra show clear voltage-dependent responses, particularly in the midto-high frequency domains, which reflect evolving charge-transfer and mass-transport resistances (Figure 3i). Additionally, EIS measurements were conducted under varying oxygen partial pressures to differentiate between ORR and the water oxidation reaction at the dual-layer PNC electrode. Distribution of relaxation times (DRT) analysis was used to identify electrochemical subprocesses.^32,36,53^ The EIS spectra, in the frequency range of 10^6^–10^–1^ Hz, revealed distinct peaks: low-frequency (LF, 10^–1^–10^2^ Hz) related to gas diffusion and surface adsorption/desorption, midfrequency (MF, 10^2^–10^4^ Hz) corresponding to bulk diffusion and surface oxygen exchange, and high-frequency (HF, 10^4^–10^6^ Hz) associated with charge transfer. The area under each peak correlates with the polarization resistance of the respective process. As shown in Figure 3j, as the oxygen partial pressure (pO_2_) increases, the midfrequency (MF) DRT feature, assigned to surface exchange and charge transfer, shifts to higher characteristic frequencies and decreases in amplitude, indicating faster interfacial kinetics. The mixed-conducting interlayer mitigates the kinetic penalty at the electrolyte junction, damping the pO_2_ sensitivity of the MF process relative to a single-layer configuration. Conversely, as pO_2_ decreases, the low-frequency (LF) feature, associated with gas-phase and porous transport limitations, grows in amplitude and shifts to longer relaxation times, reflecting stronger concentration polarization within the outer layer’s pore network. As pO_2_ increases from 10% to 30%, the MF peak shifts from log *f* = 2.2 to 2.9, indicating faster surface exchange. The LF peak shows a concurrent 33% reduction in area, consistent with suppressed gas-diffusion impedance. The dual-layer electrode demonstrates clear advantages over the single-layer design, including improved interfacial bonding, enhanced surface properties, and greater long-term stability. DRT analysis further confirms accelerated gas diffusion, faster surface exchange, and more efficient charge transfer. These results underscore the importance of hierarchical microstructural engineering in optimizing electrode performance.

### 2.3 Improved Electrochemical Performances in Fuel Cell and Steam Electrolysis Modes

The electrochemical performance of full cells utilizing dual-layer PNC electrode was assessed and compared to single-layer electrode in both fuel cell and electrolysis modes under various experimental conditions. Two distinct cells were fabricated: one with a conventional powder-packed electrode and the other featuring a dual-layer PNC electrode, to emphasize the improvements offered by the new structure. The open-circuit voltages of both cells were above 1.03 V, confirming a dense electrolyte membrane and the absence of gas leakage (Figure 4a,b). The dual-layer cell demonstrated a 40% increase in peak power density (PPD) compared to the single-layer PNC cells, achieving an optimized PPD of 0.955 W cm^–2^ at 600 °C with H_2_ (20 sccm) and O_2_ (40 sccm) fed to the electrodes separately. This performance surpasses the results reported in recent literature. At the tested temperature, the PPD increased by 61%, 24%, and 16%. These results underscore the enhanced electrochemical performance of the dual-layer configuration compared to single-layer electrodes, especially in high-demand environments.

**Figure 4:**
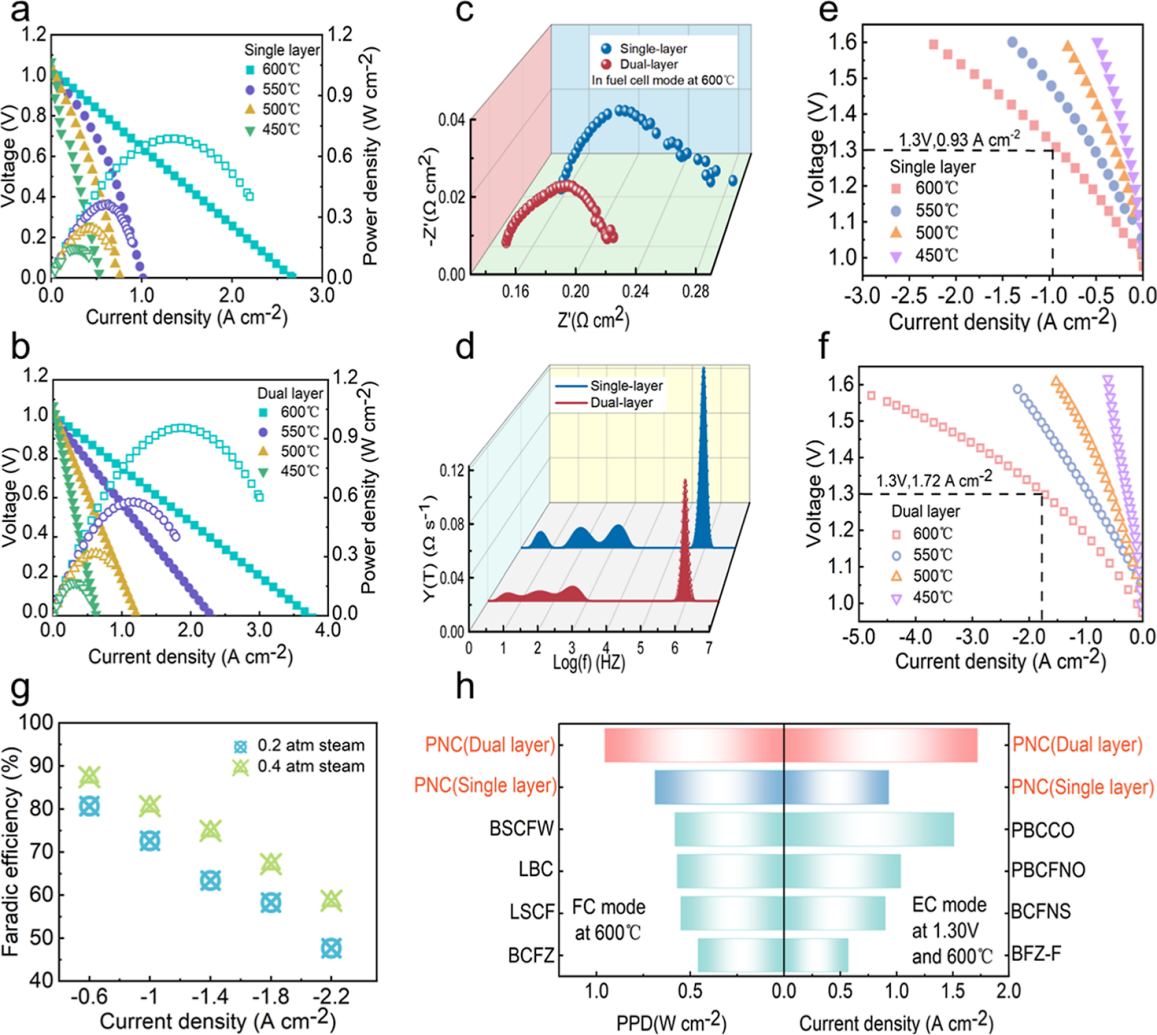
Electrochemical performance of full cells equipped with a nanoparticle-engineered dual electrode. Compared with a conventional microscale single-layer electrode, demonstrating the critical role of the nanoscale bridging interlayer in fuel cell and electrolysis operation. (a) *I*–*V*–*P* curves of the cell with a microscale single-layer PNC electrode at 450–600 °C, H_2_ (20 sccm) and O_2_ (40 sccm). (b) *I*–*V*–*P* curves of the cell incorporating a nanoscale particle-based bridging layer at 450–600 °C, H_2_ (20 sccm) and O_2_ (40 sccm). (c) EIS spectra comparison between single-layer and nanobridged dual-layer electrodes at 600 °C. (d) Corresponding DRT analysis of the EIS data. (e) Polarization curves of the single-layer electrode in electrolysis mode at 450–600 °C under H_2_ (20 sccm) and 20 vol % H_2_O + 80 vol % O_2_ (40 sccm). (f) Polarization curves of the nanobridged dual-layer electrode in electrolysis mode at 450–600 °C under the same gas condition. (g) Faradaic efficiency behaviors of the cell with dual-layer electrode under different steam pressure (20 vol % vs 40 vol % H_2_O). (h) Comparisons of PPD and CD of this nanobridged dual-layer electrode at 1.30 V and 600 °C with some benchmarking start-of-art electrodes.

The spectra measurements were performed at 600 °C to investigate the electrochemical responses to reveal the underlying mechanism. The Nyquist plots (Figure 4c) show that the dual-layer electrode exhibits a smaller semicircular arc in the high-frequency region, indicating reduced charge transfer resistance compared to the single-layer electrode. Comparison of the impedance collected at different temperatures (600–450 °C, Figures S20 and 21, Supporting Information) further confirms that both ohmic resistance and electrode polarization resistance are reduced, which reflects the enhancement of charge transfer and electrode kinetics simultaneously at interface. To gain deeper insights into electrochemical dynamics at 600 °C, the EIS data were subjected to DRT analysis, as illustrated in Figure 4d. This analysis involved separating the three impedance arcs and fitting the corresponding parameters, which enabled the identification of the underlying electrochemical processes. The dual-layer electrode under FC mode displays reduced LF intensities compared to the conventional structure, suggesting that the bridging architecture effectively enhances mass transport. In addition, the further suppression of MF peaks confirms a more efficient oxygen surface exchange, involving accelerated adsorption, dissociation, and incorporation at the interface.

In electrolysis mode, the dual-layer electrode demonstrated favorable performance for hydrogen production, particularly when compared to the current densities observed in single-layer electrode over the temperature range of 450–600 °C. As depicted in Figure 4e,f, both electrode configurations exhibit typical electrolysis characteristics, where voltage increases with rising current density, followed by a sharp rise in voltage at higher current densities due to polarization losses. At 600 °C, the electrolysis current density for the dual-layer electrode reached 2.85 A cm^–2^ at 1.4 V, compared to only 1.39 A cm^–2^ for the single-layer cell, representing a 105% improvement in electrolysis performance. Comparison of the EIS spectra at different temperatures when the cells were operated at 1.30 V reveals the reductions of ohmic and polarization resistances in the dual-layer design (Figures S22 and S23, Supporting Information). These results confirm that the dual-layer structure enhances electrode kinetics and reduces total resistance, contributing to improved performance in both fuel cell and electrolysis modes. The consistently lower overpotentials across the temperature range reflect more efficient ion/electron transport and increased reaction kinetics.

Faradaic efficiency, a key parameter for evaluating the efficiency of electrochemical processes, was measured for the dual-layer electrode structured cells under different steam concentrations and electrolysis current densities. As shown in Figure 4g, specifically, the Faradaic efficiency is measured at 80.66% under the 20 vol % H_2_O + 80 vol % O_2_ mixture, and 87.34% under the 40 vol % H_2_O + 60 vol % O_2_ mixture. These results demonstrate that an increase in the water content ratio leads to a higher Faradaic efficiency. To benchmark against state-of-the-art oxygen electrodes in PCCs, the PPD in fuel cell mode and electrolysis current density (CD) at a thermoneutral voltage of 1.30 V and 600 °C were compared. The results, presented in Figure 4h, clearly demonstrate that the dual-layer PNC electrode achieves higher PPD and CD across a wider range of current densities, outperforming the single-layer electrodes in all tested conditions. The dual-layer PNC electrode demonstrates outstanding electrochemical performance, delivering higher peak power density and electrolysis current density across a wide range of operating conditions.^18,34,54–58^ This superior performance underscores their ability to sustain high efficiency in both fuel cell and electrolysis modes, even under thermoneutral voltage operation. Compared to other state-of-the-art oxygen electrodes in PCCs, the dual-layer design exhibits notable advantages in charge transfer kinetics and Faradaic efficiency.

### 2.4 Robust Durability of Electrochemical Performance under Various Dynamic and Reversible Operation Conditions

Figure 5a shows a short-term electrolysis test comparing single-layer and dual-layer electrodes, conducted at a constant voltage of 1.4 V and 600 °C over 70 h. The cell with the single-layer electrode showed a considerable decline in electrolysis current density, decreasing from 1.15 to 0.62 A cm^–2^, likely due to interfacial degradation resulting from insufficient interface optimization. In contrast, the dual-layer electrode maintained a much more stable current density throughout the test, indicating improved interfacial integrity and sustained reaction kinetics under prolonged electrolysis conditions. Figure 5b displays the results of transient electrolysis tests, in which the applied voltage was alternated between 1.30 and 0.70 V, and between 1.50 and 0.50 V. Each voltage cycle comprising an increase followed by a return to the original value was maintained for 1 h and repeated 20 times. These dynamic tests, conducted at 600 °C, were designed to simulate the voltage fluctuations commonly encountered in practical electrochemical systems due to varying load conditions and environmental changes. By incorporating both fuel cell and electrolysis operating modes, the tests more accurately reflect real-world operational stresses. Under these conditions, the dual-layer PNC electrodes exhibited superior durability and resistance to performance degradation, due to the enhanced interfacial stability and optimized transport properties afforded by the dual-layer architecture. The long-term durability of a PCC is dependent on many factors, in which the compositions, microstructures, interfaces, and interlayer diffusion are some primary elements. Under high steam condition, the interfacial durability becomes a dominant factor when the composition is well optimized. Therefore, examining stability under some extreme conditions is very critical to validate this dual-layer electrode’s feasibility in operating at various dynamic, reversible conditions. Stability tests in fuel cell (FC) mode are shown in (Figures S24 and S25, Supporting Information), while Figure S26 presents the EIS spectra obtained before and after the transient cycling in fuel cell mode.

**Figure 5:**
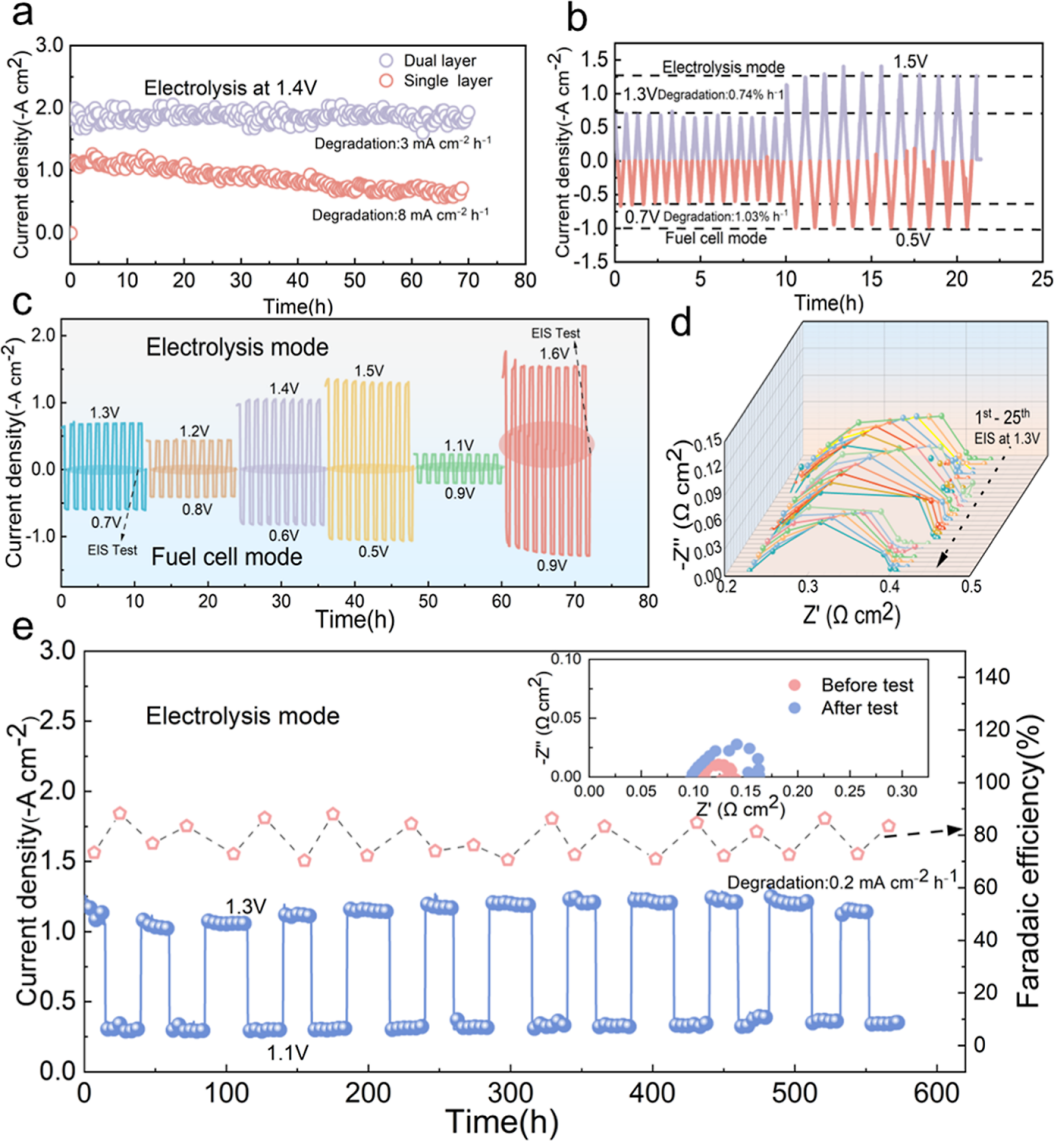
Enhanced durability of full cells with nanobridged dual electrode under various electrolysis operation conditions. (600 °C, H_2_ (20 sccm) and O_2_ (20 sccm)) (a) Electrolysis current density stability of the cells with single- and dual-layer electrodes at 1.4 V and 600 °C. (b) Reversible voltage cycling between 1.30 and 0.70 V and 1.50–0.50 V, with each cycle lasting 1 h, simulating dynamic operation. 20% H_2_O was supplied with oxygen into steam electrode side. (c) Step-voltage transient tests under alternating fuel cell and electrolysis modes. Applied voltage ranges included 1.30–0.70, 1.20–0.80, 1.40–0.60, 1.50–0.50, 1.10–0.90, and 1.60–0.40 V to evaluate performance stability under variable conditions. (d) EIS spectra collected at 1.30 V over 25 cycles during long-term stability testing, highlighting the evolution of electrode impedance and degradation behavior. (e) The long-term electrolysis stability and Faradaic efficiency testing.

In addition to stepwise cycling, the applied potential was alternated across multiple voltage intervals: 1.30–0.70 V, 1.20–0.80 V, 1.40–0.60 V, 1.50–0.50 V, 1.10–0.90 V, and 1.60–0.40 V—to evaluate electrochemical stability under a broad range of dynamic operating conditions. The full cell was continuously operated at 20% H_2_O at 600 °C for a total of 74 h (Figure 5c). At each voltage interval, the dual-layer PNC electrode maintained stable electrolysis current densities with negligible performance decay (e.g., from 1.35 to 1.30 A cm^–2^), whereas single-layer counterparts exhibited progressive degradation. Supporting data in Figures S27 and S28 further confirm the structural and electrochemical resilience of the dual-layer configuration across all tested voltage regimes. During the testing, the EIS measurements were carried out to monitor the in situ change of the cell resistances. Figure 5d shows the spectra collected at 1.30 V over 25 cycles during the durability test. The dual-layer electrode exhibited stable ohmic resistance (*R*_o_ ≈ 0.21 Ω cm^2^) and polarization resistance (*R*_p_ ≈ 0.198 Ω cm^2^), indicating excellent electrochemical stability over prolonged operation. Under step switching between 1.1 and 1.3 V at *p*H_2_O = 0.2 atm, the cell sustains electrolysis-mode operation for 573 h, with the current density reproducibly toggling between ∼0.3 A cm^–2^ (1.1 V) and ∼1.1–1.2 A cm^–2^ (1.3 V) while the Faradaic efficiency remains near ∼85% (Figure 5e). A linear fit to the time trend yields an average degradation rate of ∼0.2 mA cm^–2^ h^–1^. Electrochemical impedance spectroscopy (EIS) collected before and after the long-term test shows an increase in polarization resistance from *R*_p_ = 0.0293 to 0.0629 Ω cm^2^ with a nearly unchanged high-frequency intercept, indicating stable ohmic resistance and a robust electrode/electrolyte interface. The Faradaic efficiency shortfall suggests opportunities for optimization, potentially related to side reactions, gas crossover, or mass-transport limitations. (Figure 5e).

### 2.5 Faradaic Efficiency and Electrochemical Performance of the PCCs under Harsh Operating Conditions

Figure 6 illustrates the electrochemical performance stability of the cell using a dual-layer PNC electrode operated at 600 °C under varying current densities and steam partial pressures. The results provide insight into long-term voltage behavior, Faradaic efficiency, and operational resilience under both steady-state and transient conditions. As shown in Figure 6a,b, we first stepped the electrolysis current density from −0.6 to −2.2 A cm^–2^ at *p*H_2_O = 0.2 atm for 36 h. We then raised *p*H_2_O to 0.4 atm and stepped the current back from −1.8 to −0.6 A cm^–2^ over the next 28 h. Finally, stability under dynamic operation was assessed for 80 h at 0.2 atm by square-wave cycling between −1.8/–0.6 and −1.4/–1.0 A cm^–2^. Faradaic efficiency (FE) was high across most steady states: at 0.2 atm it peaked at 80% at −0.6 A cm^–2^ and decreased to 48% at −2.2 A cm^–2^, whereas at 0.4 atm it improved to 88% at −0.6 A cm^–2^ and declined to 67% at −1.8 A cm^–2^ (Table S14, Supporting Information). The FE gain with higher *p*H_2_O is consistent with humidity-driven defect equilibria that raises protonic defect concentration and suppress hole-mediated electronic leakage, shifting the overall charge-transport balance in the electrolyte. During transient cycling, FE exhibited noticeable fluctuations, most pronounced in the lower-current window (−1.0 to −1.4 A cm^–2^) at 0.2 atm, arising from the time needed for hydration, surface adsorption/dissociation, and gas-phase transport to re-equilibrate after a step change; in drier conditions these processes relax more slowly and are more susceptible to local steam depletion and short-time redox/reconstruction at the oxygen electrode. Under sustained operation at 600 °C and *p*H_2_O = 0.4 atm (Figure 6b), the cell maintained stable performance over 140 h. The H_2_ production rate scaled linearly with current density, while FE remained mostly >70% but gradually decreased at higher current density, attributable to larger overpotentials and a growing parasitic leakage fraction under high load. These results highlight both the beneficial role of elevated steam pressure in stabilizing FE and the need for electrode architectures that minimize transient lag and parasitic pathways under fluctuating demand.

**Figure 6:**
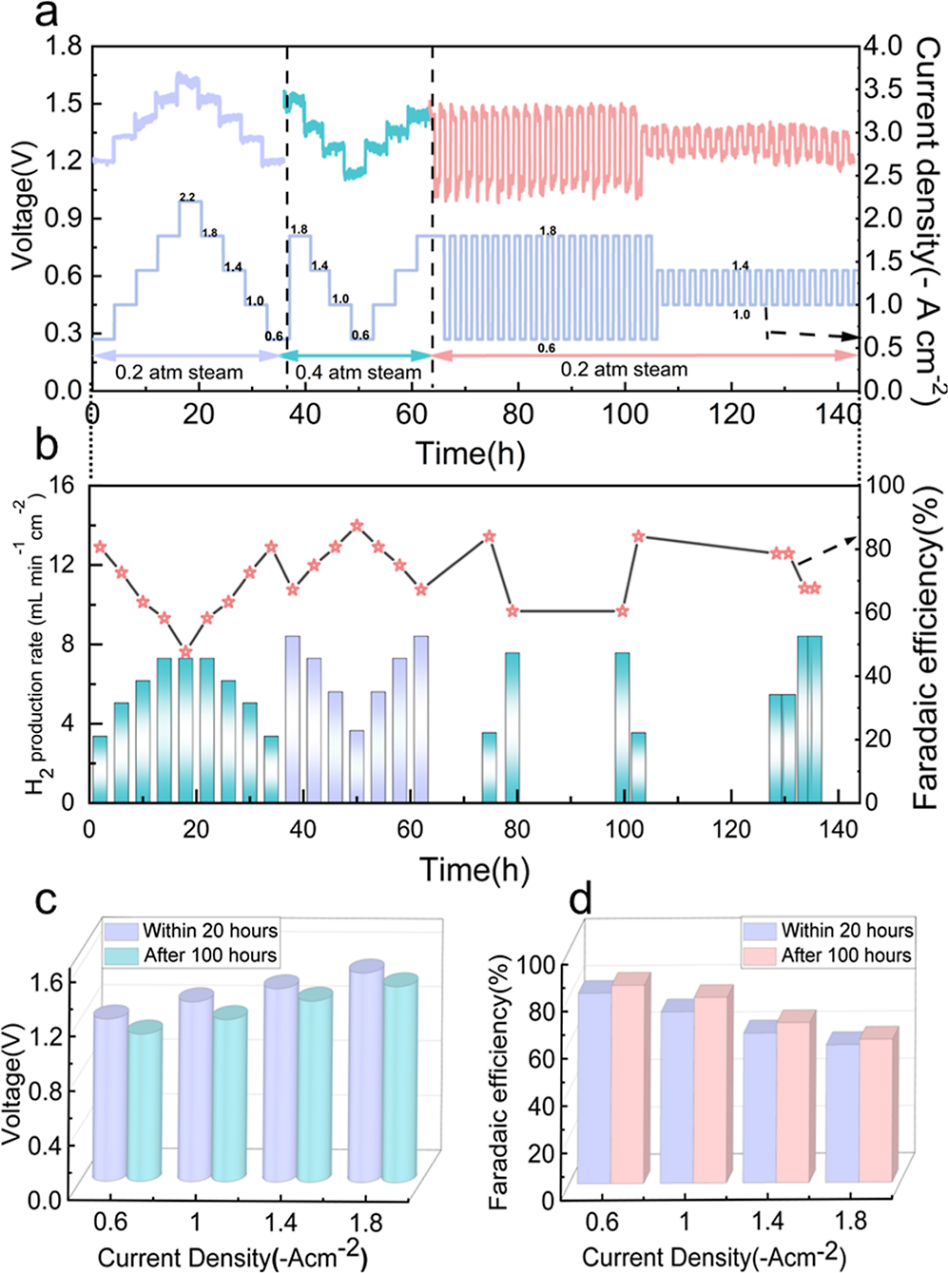
Electrochemical performance durability of the PCCs under high current densities and steam pressure. (a) Specifically, the cell was operated at −0.6, −1.0, −1.4, −1.8, and −2.2 A cm^–2^ under 0.2 atm H_2_O for 36 h; followed by −1.8, −1.4, −1.0, and −0.6 A cm^–2^ under 0.4 atm H_2_O for 28 h. Additionally, transient tests involving cyclic current variations from −1.8 to −0.6 A cm^–2^ and from −1.4 to −1.0 A cm^–2^ were conducted at 0.2 atm H_2_O for a total of 80 h to assess operational stability. (b) Hydrogen evolution kinetics and Faradaic efficiency at 600 °C under a range of applied current densities. (c) Cell voltage was monitored over 80 h at different applied current densities (d) Faradaic efficiency was monitored over 80 h at different applied current densities.

The voltage responses of the electrochemical cell at various current densities (−0.6 to −1.8 A cm^–2^) are shown in Figure 6c, with measurements taken within the initial 20 h (purple) and after 100 h of continuous operation (green). The progressive voltage drop observed across all current densities is indicative of improved electrochemical activation and reduced interfacial resistance, suggesting enhanced system performance during operation. Nonetheless, the voltage remains relatively stable, particularly under low to moderate current loads, suggesting good electrochemical durability over extended periods. Figure 6d summarizes the corresponding Faradaic efficiencies, revealing an interesting trend: instead of decreasing, the Faradaic efficiency values show a slight increase after 100 h of operation, particularly under low to moderate current densities. This improvement may be attributed to enhanced electrode–electrolyte interface contact, improved electrode activation, or reduced side reactions over time. The observed rise in Faradaic efficiency indicates not only the robustness of the cell architecture, but also its increasing electrochemical utilization efficiency with extended operation, highlighting the system’s potential for stable long-term performance.

During transient cycles, both voltage and Faradaic efficiency showed oscillations, highlighting delayed system response to rapid current changes. These fluctuations underscore the need to optimize dynamic operation for stability under variable loads. While the system performs reliably in a steady state, abrupt changes in current density introduce temporary inefficiencies. Overall, reducing transient fluctuations is crucial to sustaining high efficiency and long-term stability in proton-conducting electrochemical cells, particularly in dynamic applications. Further research is essential to identify strategies for mitigating transient performance losses and improving the operational stability of the system under fluctuating load conditions.

As shown in Figure S30 and S31 (Supporting Information), the cross-sectional view of the cell displays a robust four-layer structure consisting of the cathode, electrolyte, AFL, and anode support. The dense, pinhole-free electrolyte (5–10 μm thick) effectively prevents fuel crossover while maintaining structural integrity. At the electrolyte/AFL interface (Figure S30b), complete reduction to metallic nickel in the anode support is observed, which promotes proton transport and lowers interfacial resistance, thereby enhancing overall fuel cell performance. High-magnification imaging (Figure S30c) further reveals a seamless interface between the PNC electrode and the electrolyte, ensuring efficient electron/ion transfer during ORR. The porous architecture of the anode support facilitates hydrogen diffusion, supporting long-term stable operation. Finally, Figure S30d highlights the smooth, uniform surface morphology of the electrolyte, which minimizes parasitic reactions and contributes to durability.

### 2.6 Fundamental Mechanisms Explaining Why Nanoparticles Promote Sintering and Enhance Interfacial Electrochemical Performance

Nanoparticles promote sintering and enhance interfacial electrochemical performance primarily due to their high per unit volume surface energy. The surface energy of a single particle is directly proportional to its surface area, which increases with the square of the particle radius. Mathematically, the total surface energy (*E*_surface)_ of a spherical particle can be expressed as^59^

2 $$E_{surface}=\gamma \cdot A=\gamma \cdot 4\pi R^{2}$$

where γ is the surface energy per unit area, and *A* is the surface area of the particle. Thus, for an individual particle, *E*_surface_ increases with *R* ^2^. This statement applies only to one particle. For sintering and interfacial electrochemical processes, the relevant driving factors are the surface energy per unit area γ together with curvature (higher for smaller *R*), or equivalently the surface-to-volume ratio *A*/*V*, rather than the absolute total surface energy of an isolated particle. In electrode materials, it is therefore more appropriate to discuss surface energy on a per-volume (or per-mass) basis, scaling with *A*/*V*, as this captures the collective contribution of all particles within a given volume and better reflects their influence on particle–electrolyte interactions, electrochemical performance, and stability.

The surface energy per unit volume is a critical parameter that characterizes the energy density associated with the surface atoms of a material. As the particle size decreases, the surface area-to-volume ratio increases, leading to a higher surface energy per unit volume. This relationship can be expressed as^60^

3 $$V=\frac{4}{3}\pi R^{3}$$

4 $$\frac{E_{surface}}{V}=\frac{4\pi R^{2}\gamma }{\frac{4}{3}\pi R^{3}}=\frac{3\gamma }{R}$$

*R* is the particle radius, and *V* is the particle volume. As the particle radius (*R*) decreases, the surface energy per unit volume increases inversely, highlighting the increased reactivity and chemical potential of smaller particles (Figure 7b).

**Figure 7:**
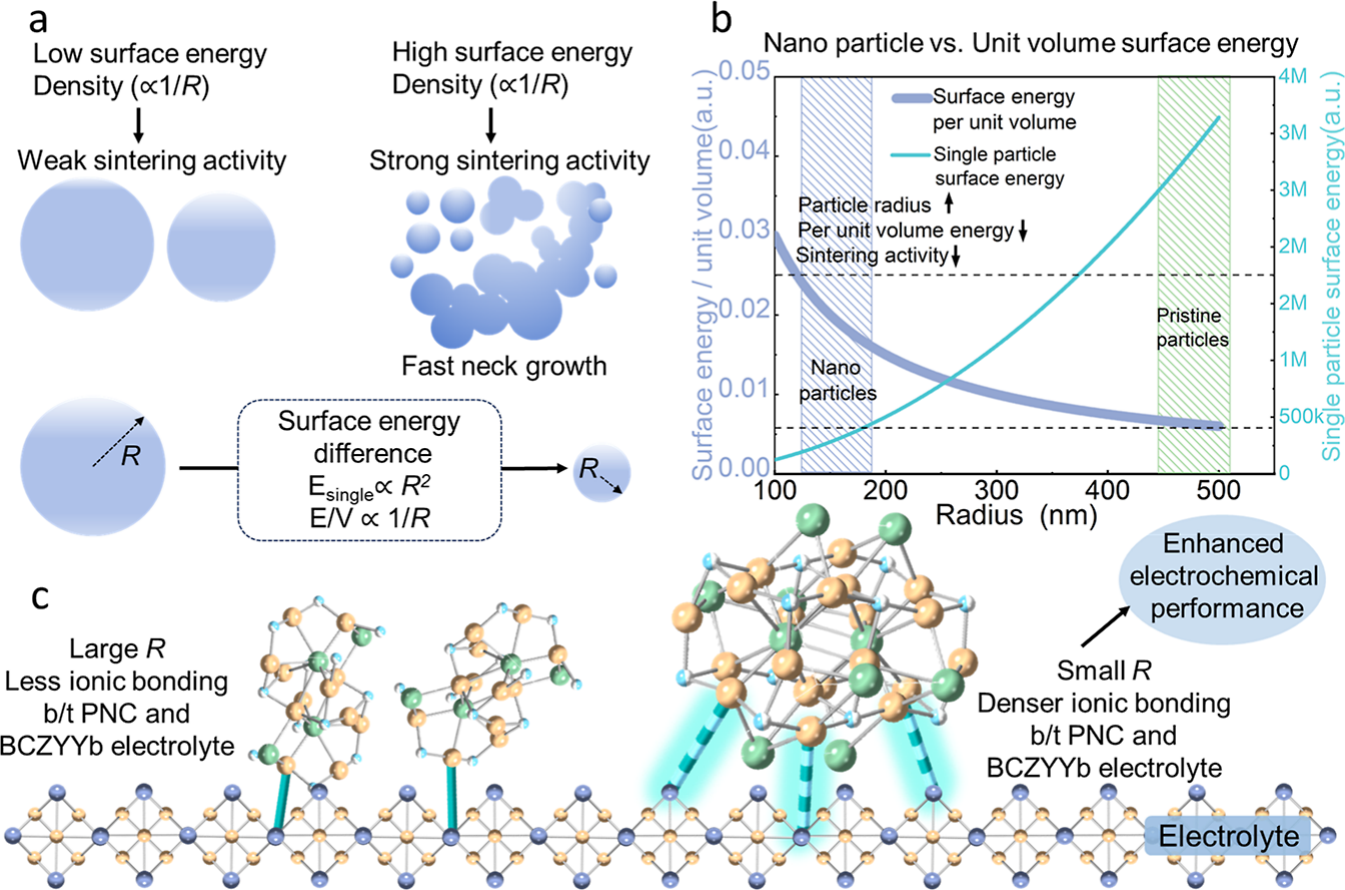
Size-dependent sintering behavior of nanoparticles: smaller nanoparticles enhance sintering and interfacial bonding, improving electrochemical performance. (a) Schematic showing that smaller nanoparticles, due to their higher surface energy, exhibit stronger sintering and faster neck growth than larger particles. (b) Surface energy increases with decreasing nanoparticle radius, revealing a pronounced size effect on sintering driving force. (c) At the electrolyte interface, reduced nanoparticle size enhances bonding, leading to improved electrochemical performance.

Using the measured radius for large and small populations (*R*_L_ = 481 nm, *R*_s_ = 168 nm), sintering kinetics and interfacial electrochemistry are governed by the surface-energy density of the ensemble (Table S15, Figure S32, Supporting Information). The surface-energy density increases inversely with size.

5 $$\frac{{\left(E/V\right)}_{S}}{{\left(E/V\right)}_{L}}=\frac{R_{L}}{R_{S}}\approx 2.895$$

In contrast, the small-particle mode exhibits 2.859 times higher than that of the large-particle mode. This scaling relationship explains the observed trends (Figure 7b): mixtures enriched in the nanoparticle mode undergo faster sintering, achieve greater bonded coverage at the interface, and consequently display reduced interfacial polarization resistance.

This higher surface energy density from nano fine particles is one of the primary reasons for their enhanced sintering behavior and improved interface bonding with electrolytes. Fine nanoparticles have a greater number of exposed atoms per unit volume, which results in stronger interatomic forces at the particle surfaces. This leads to more efficient densification and better electrochemical performance in materials such as electrodes.

Figure 7a demonstrates that nanosized particles possess a much higher surface energy density than larger grains, which significantly enhances their sintering activity. As a result, necks between adjacent nanoparticles form more rapidly, leading to a denser and more uniform microstructure during electrode fabrication. This microstructural feature is critical for ensuring good electronic and ionic connectivity throughout the electrode. The theoretical basis for this behavior shows the relationship between particle radius and surface energy (Figure 7b). Although the total surface energy of a single particle decreases with increasing size, the surface energy per unit volume increases sharply at the nanoscale. This elevated energy density acts as a strong thermodynamic driving force for sintering and interfacial consolidation, thereby accelerating densification and improving electrode integrity.

The total bonding force (*F*_total_) is directly proportional to the number of bonds (*n*) formed between atoms at the interface. The total bonding force increases linearly with the number of bonds, as described by the relation: *F*_total_ ≈ *n*·*F*_bond_, *F*_bond_ is the bonding force of a single bond. For original particles, fewer bonds are typically formed due to the lower number of active surface sites, leading to a lower total bonding force. In contrast, fine nanoparticles exhibit a greater number of surface atoms, resulting in a higher number of bonds and consequently a higher total bonding force. This relationship suggests that fine nanoparticles, with their increased bonding interactions, are more likely to form strong interfaces with the electrolyte, thus enhancing the sintering and electrochemical properties compared to original particles. Figure 7c further illustrates the atomic-scale mechanism: smaller particles enhance the ionic bonding at the electrode/electrolyte interface, which facilitates charge transfer and reduces interfacial resistance. When these nanosized particles are integrated into a bilayer electrode structure, the top layer composed of fine particles provides efficient ionic conduction and active interfacial sites, while the underlying layer maintains mechanical robustness and structural stability. Collectively, these synergistic effects explain why the nanolayer electrode constructed with nanosized particles exhibits superior electrochemical performance compared with its coarse-particle counterpart.

## 3 Conclusions

In summary, we developed a dual-layer PNC electrode architecture that leverages nanoscale interface engineering to overcome critical interfacial and performance challenges in PCCs. By employing a fine-grained, nanoparticle-derived interfacial contact layer beneath a catalytically active porous backbone, this design promotes dense nanoscale sintering with the BCZYYb electrolyte, leading to strong chemical bonding, enhanced adhesion strength, and continuous ion/electron transport across the interface. This architecture effectively suppresses interfacial degradation under both thermal and electrochemical cycling, enabling stable operation across diverse dynamic conditions. As a result, the dual-layer electrode delivers a 40% improvement in peak power density and a 130% increase in electrolysis current density, while sustaining high Faradaic efficiency of up to 88% under elevated steam concentrations. These findings highlight nanoengineered interfaces as crucial for next-generation PCCs, where interfacial cohesion and reaction kinetics must be jointly optimized for high performance and durability. Beyond validating the dual-layer structure, this work establishes a generalizable nanoarchitectural strategy integrating catalytic activity, mechanical integrity, and scalability. Future efforts in tailoring nanoscale composition gradients, adaptive interface chemistries, and long-term cycling under realistic conditions will further advance this approach. The demonstrated reproducibility and durability of the dual-layer electrode make it a strong candidate for reversible fuel cells and high-efficiency hydrogen production.

## 4 Methods

### 4.1 Materials Synthesis

PrNi_0.7_Co_0.3_O_3‑δ_ powders were synthesized via a sol–gel combustion method. First, EDTA was dissolved in deionized water under stirring (350–450 rpm) at room temperature, with ammonia added to adjust the pH to 7–8 until the solution was clear. Pr­(NO_3_)_3_·6H_2_O (99.9%), Ni­(NO_3_)_2_·6H_2_O (99%), and Co­(NO_3_)_2_·6H_2_O (99.9%) were sequentially added, with the pH was readjusted to 7–8. After stirring for 10–15 min, citric acid was added (EDTA: CA: metal cation molar ratio = 1:1.5:1), and the mixture was stirred for 30 min until fully transparent. The solution was heated to 300 °C to evaporate water and form a dark gel, which expanded and auto ignited, yielding a black ash. The ash was calcined at 1000 °C for 5 h to obtain crystalline PNC powder. For pristine PNC, the calcined powder was ball-milled with 2 wt % ethyl cellulose in terpineol/ethanol for 30 min. For PNC nanoparticles, the same mixture was ball-milled for 24 h to reduce particle size. Specifically, we used a planetary ball mill (model PPMV1–1L, MSE Supplies LLC, USA) equipped with a 500 mL ZrO_2_ jar and ZrO_2_ grinding media (5.5 mm and 10 mm in diameter) at a ball-to-powder mass ratio of 5:1. The precursors were mixed in ethanol (solids ≈99.9 wt %) and milled at 300 rpm for 24 h, with a 30 min rest after every 180 min to minimize thermal buildup.

The BCZYYb electrolyte powders were synthesized via a sol–gel method. (Supporting Information Materials synthesis)

### 4.2 Fabrication of Cells

Symmetric cells were fabricated by uniaxially pressing BCZYYb powder (containing 2 wt % NiO) into pellets, followed by sintering at 1450 °C for 10 h in ambient air to ensure full densification. Pristine or fine PNC particle slurries were brush-coated onto both sides of the pellet (active area: 0.178 cm^2^) and calcined at 1000 °C for 5 h to remove organic binders, yielding the final symmetric cells.

Fuel cell: first, we weighed NiO, BCZYYb, and starch at 3:2:1 wt %, mixed and ground them in an agate mortar for 30 min to ensure homogeneity; next, a NiO–BCZYYb (3:2 wt %) mixture was designated as the functional interlayer and pure BCZYYb as the electrolyte; then the powders were sequentially loaded into a 12 mm steel die­(anode (0.40 g) → anode functional layer (0.010 g) → electrolyte (0.004 g) with the smaller electrolyte mass chosen to yield a thin layer). After that, we uniaxially pressed the trilayer green body at 100 psi for 1 min; subsequently, the half-cell pellets were sintered at 1450 °C for 10 h in ambient air to obtain a dense electrolyte; finally, we brush-coated PNC slurry (pristine or nanoparticle-based) onto the electrolyte surface (active area 0.178 cm^2^), then calcined at 1000 °C for 5 h in ambient air to remove organic binders.

### 4.3 Cells Assembly and Electrochemical Measurements

The cells were mounted onto custom-made test fixtures (Figure S29, Supporting Information) and sealed with Ceramabond 552, with the oxygen electrode oriented upward. A silver mesh served as the current collector and was connected to silver wires. Specifically, the Ag mesh was fabricated by winding silver wire (Thermo Fisher Scientific, Ward Hill, MA, USA; 0.25 mm diameter, 99.9% metals basis, Lot: P03L013) into a grid. The mesh was then attached to the electrode using silver conductive adhesive paste (Thermo Fisher Scientific, Lot: N14L042). A thin layer of the paste was applied with a fine brush to cover the mesh–electrode interface uniformly, followed by drying at 150 °C for 2 h to ensure good electrical and mechanical contact. The assemblies were heated to 600 °C at 2 °C min^–1^ under 20 sccm H_2_ to the hydrogen electrode, enabling in situ reduction of NiO to metallic Ni. Once the open-circuit voltage (OCV) stabilized, indicating complete reduction, 40 sccm O_2_ was introduced to the oxygen electrode, and electrochemical measurements were performed using a potentiostat (Parstat MC, Princeton Applied Research). In fuel cell mode, full cells were characterized by recording current–voltage–power (*I*–*V*–*P*) curves and EIS at OCV condition over a frequency range of 10^6^ to 0.01 Hz, across 600–450 °C. In steam electrolysis mode, humidified oxygen was supplied to the cathode via a custom-designed humidifier, with precise flow control ensured by multiple flow meters. Polarization curves and impedance spectra were collected at 1.30 V within the same temperature range. Advanced impedance data interpretation was carried out using DRT analysis in Gamry Echem Analyst software, and activation energies were determined using the Arrhenius relationship. Operational durability at 600 °C was assessed through: (1) long-term stability test under 1.40 V for 70 h; (2) transient cycling between 1.30/0.70 V and 1.50/0.50 V with 1 h per cycle; and (3) stepwise voltage switching across 1.30, 0.70, 1.20, 0.80, 1.40, 0.60, 1.50, 1.10, 0.90, 1.60, and 0.40 V to evaluate stability under dynamic fuel cell/electrolysis operation. During all tests, H_2_ was supplied to the anode at 20 sccm. The cathode received either dry O_2_ (fuel cell mode) or 20 vol % H_2_O + 80 vol % O_2_ (electrolysis mode), both at 40 sccm. Reversible operation between operating modes was performed under these conditions. All data were collected and processed using Versa Studio software.

### 4.4 Structure Characterizations

A scanning electron microscope (TFS Quattro S) was employed to examine the cross-sectional morphology of single-layer and dual-layer PNC electrodes in both secondary electron (SE) and backscattered electron (BSE) modes. The BSE imaging revealed the detailed ultraporous architecture of the dual-layer PNC electrode in the full cell. Complementary surface topography analysis using atomic force microscopy (AFM, Park NX10) further corroborated these observations. While the electrolyte surface exhibited a relatively smooth profile, the fine-particle interfacial layer of the dual-layer cathode displayed markedly higher surface roughness, indicative of increased surface area and enhanced potential for electrode–electrolyte interaction.

### 4.5 Measurement of Electrode Bonding Strength

Prepare Sample A by bonding the test specimen to one end of a stainless steel or aluminum plate (Substrate 2) using cyanoacrylate adhesive, allowing it to cure for at least 12 h to ensure full adhesive strength. For Sample B, apply double-sided adhesive tape to one end of another plate (Substrate 1), then align and press Substrate 2 firmly onto the exposed surface of the test specimen, ensuring a uniform, bubble-free interface. Configure the MARK-10 tensile testing machine by setting the appropriate test speed and strain rate and mount the sample assembly securely so that the bonded interface is centered between the grips. Initiate the test to apply a controlled shear force across the interface while continuously recording the force–displacement (*F*–*S*) or force–time (*F*–*T*) response curve.

## Supplementary Material



## Data Availability

The data that support the findings of this study are available from the corresponding author upon the reasonable request.

## Notes

Materials synthesis, STEM-EDS line mappings, band-shaped ROI, global thresholding (Otsu), error bar of shear strength, EIS fitting, and statistical analysis of nanoparticles (PDF)
