# Supplementary material for: Nano-Engineered Interfaces in Dual-Layer Electrodes for Protonic Ceramic Cells with Enhanced Stability and Kinetics
Source: ACS Nano. 2025 Dec 8;19(50):42566–79. doi: 10.1021/acsnano.5c15759 (PMC12752702; doi:10.1021/acsnano.5c15759)
Supplement: Supplementary file 1 [file nn5c15759_si_001.pdf]

# Supporting Information

## Nano-Engineered Interfaces in Dual-Layer Electrodes for Protonic Ceramic Cells with Enhanced Stability and Kinetics

Yuqi Geng[1,†], Shuanglin Zheng[1,†], Saroj Karki[1], Alejandro Serrano[1], Yijie Jiang[1], Bello Idris[1], Anshu Kumari[1], Dongyang Cao[1], Hanping Ding[1,*]

1. School of Aerospace and Mechanical Engineering, University of Oklahoma, Norman, OK 73019, United States

† These authors contribute equally to this work.

* Correspondence author's email: H. Ding (hding@ou.edu)

### 4.1. Materials synthesis

$PrNi_{0.7}Co_{0.3}O_{3-\delta}$ powders were synthesized via a sol-gel combustion method. First, EDTA was dissolved in deionized water under stirring (350-450 rpm) at room temperature, with ammonia added to adjust the pH to 7–8 until the solution was clear. $Pr(NO_3)_3\cdot6H_2O$ (99.9%), $Ni(NO_3)_2\cdot6H_2O$ (99%), and $Co(NO_3)_2\cdot6H_2O$ (99.9%) were sequentially added, with the pH was readjusted to 7–8. After stirring for 10–15 minutes, citric acid was added (EDTA: CA: metal cation molar ratio = 1: 1.5: 1), and the mixture was stirred for 30 min until fully transparent. The solution was heated to 300 °C to evaporate water and form a dark gel, which expanded and auto ignited, yielding a black ash. The ash was calcined at 1000 °C for 5 h to obtain crystalline PNC powder. For pristine PNC, the calcined powder was ball-milled with 2 wt.% ethyl cellulose in terpineol/ethanol for 30 min. For PNC nanoparticles, the same mixture was ball-milled for 24 h to reduce particle size. Specifically, we used a planetary ball mill (Model PPMV1-1L, MSE Supplies LLC, USA) equipped with a 500 mL $ZrO_2$ jar and $ZrO_2$ grinding media (5.5 mm and 10 mm in diameter) at a ball-to-powder mass ratio of 5:1. The precursors were mixed in ethanol (solids ≈ 99.9 wt%) and milled at 300 rpm for 24 h, with a 30-min rest after every 180 min to minimize thermal buildup.

The BCZYYb electrolyte powders were synthesized via a sol-gel method using $Ba(NO_3)_2$, Ce $(NO_3)_3\cdot6H_2O$, $ZrO(NO_3)_2\cdot xH_2O$, $Y_2O_3$, and $Yb_2O_3$ as precursors, with EDTA and citric acid as complexing agents. $Y_2O_3$ and $Yb_2O_3$ were dissolved in 20 wt.% nitric acid at 300 °C with stirring, Zr nitrate was dissolved separately in deionized water, while EDTA was dissolved and adjusted to pH ~7 using ammonia. Once the Zr and EDTA solutions were clear, they were combined, followed by sequential addition of Ba and Ce nitrates, the cooled Y/Yb solution, and citric acid. After final pH adjustment to ~7, the clear solution was heated to 300 °C to form a dark gel that self-ignites. The resulting ash was calcined at 1000 °C for 5 h to obtain phase-pure BCZYYb powder. The anode support layer was prepared by mixing NiO, BCZYYb, and corn starch in a weight ratio of 3:2:1. The functional layer was synthesized via the same sol-gel route as the electrolyte powder and mixed with NiO at a 3:2 weight ratio to form the final anode composition. The BCZYYb powders for symmetric cells were synthesized via a solid-state ball milling method using high-purity $BaCO_3$, $ZrO_2$, $CeO_2$, $Y_2O_3$, and $Yb_2O_3$ (Thermo Fisher, ≥99.5%). The precursors were mixed in ethanol and milled at 300 rpm for 24 h, then dried and calcined at 1100 °C for 10 h in air. The calcined powder was milled again under the same conditions for 12 h and subjected to a second calcination at 1100 °C for 10 h to obtain phase-pure, crystalline BCZYYb.

**Table S1**. comparing previously reported dual-layer or graded electrode structures in protonic ceramic cells.

| Cell configuration | Temperature (℃) | Power density ($W\ cm^{-2}$) | Current density at 1.3V ($A\ cm^{-2}$) | Refs. |
|---|---|---|---|---|
| NiO-ScSZ//ScSZ//GDC// LSCF | 700 | 1.33 | 1.25 | [39] |
| NiO-YSZ//YSZ//LYDC(37-42-21)// LYDC(47-42-11) | 800 | 0.72 | N/A | [40] |
| NiO–30% YSZ-rice flour// YSZ// 50%YSZ–50%LSM // LSM | 800 | 0.9 | N/A | [41] |
| Ni-BCZY//BCZ//50% PBCO-50%BCZY// 75% PBCO-25% BCZY//50%PBCO-50%BCZY | 700 | 0.588 | N/A | [42] |
| NiO-BCZY//CG-AFL// BCZY//SSC- BCZY | 700 | 0.521 | N/A | [43] |
| NiO-BCZYYb+Starch// NiO-BCZYYb//BCZYYb//Nano-PNC-Mirco-PNC | 600 | 0.96 | 1.72 | this work |

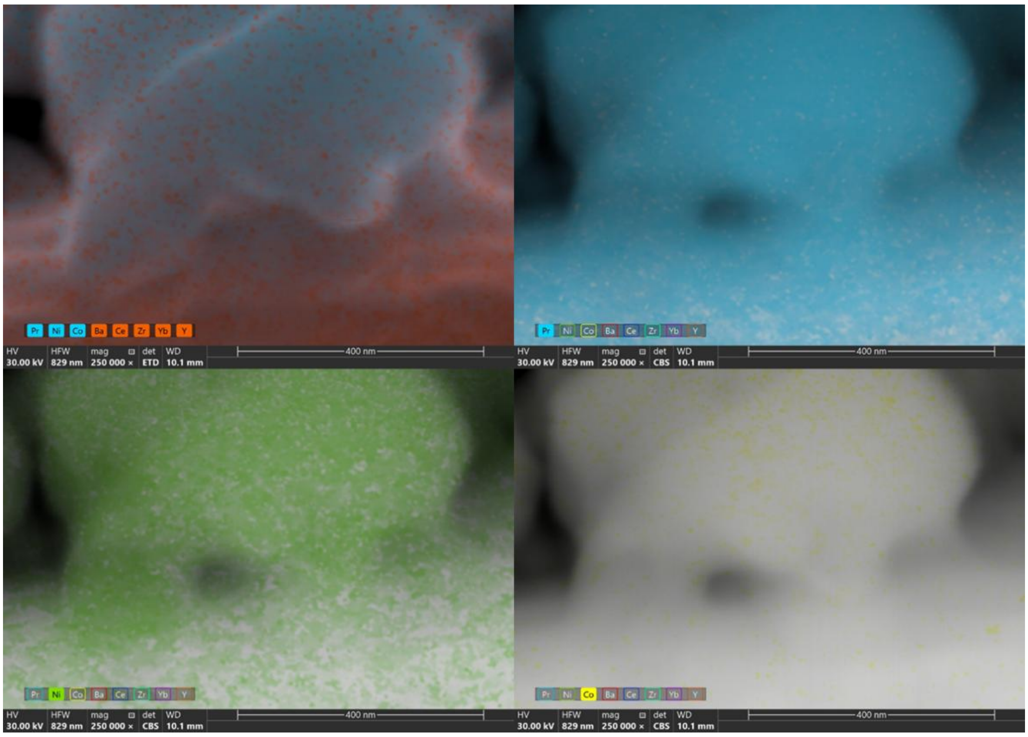


**Figure S1**. Elemental Mapping of the PNC73 Phase at the Interface with BCZYYb.

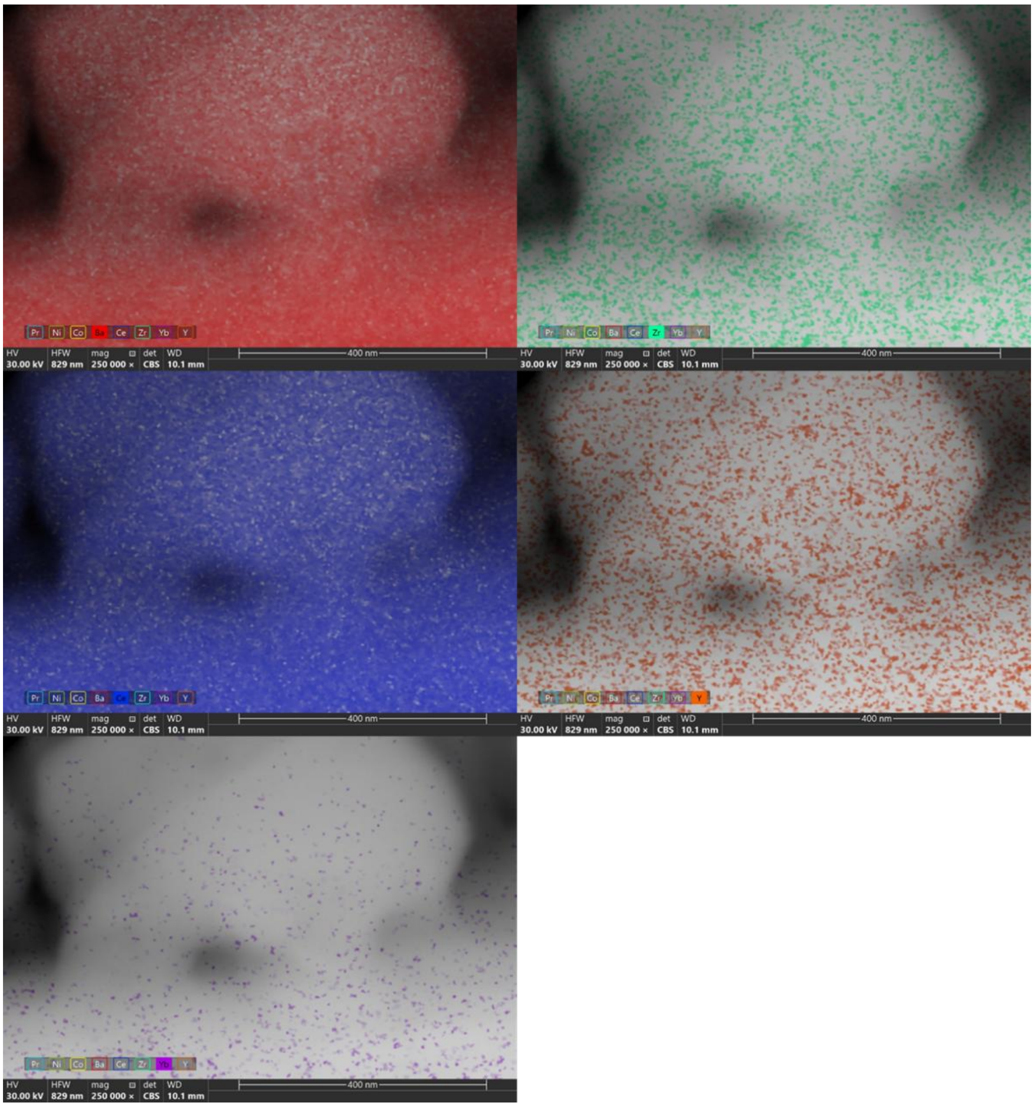


**Figure S2**. Elemental Mapping of BCZYYb to Visualize Elemental Distribution at the Interface with PNC.

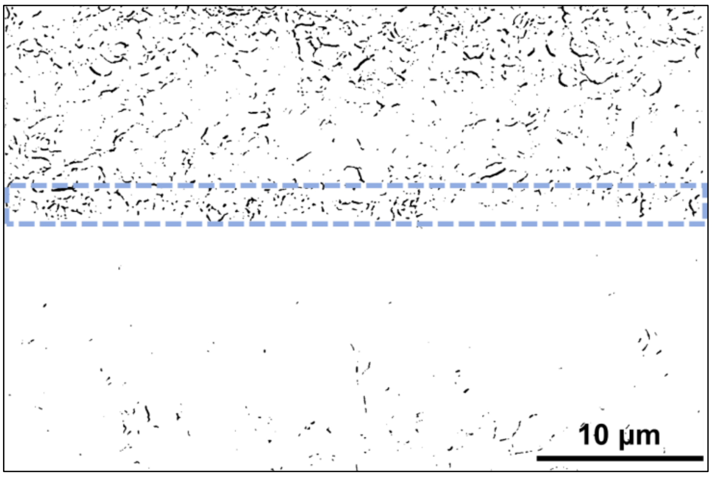


**Figure S3**. Open-porosity area fraction (pore area/ROI area)

Using pixel-calibrated cross-sectional SEM images, we quantified the interfacial porosity within a band-shaped ROI by applying background subtraction, global thresholding (Otsu), and computing the open-porosity area fraction (pore area/ROI area). This analysis yields an interfacial porosity of 4.6%. This very low value, which is substantially below that of the adjacent porous regions analyzed by the same protocol (typically >30%), indicates that the layer is well densified and forms a continuous bonding layer with minimal unbonded voids. Consequently, the effective load-bearing area is maximized, and the defect density is low, supporting the conclusion that the interfacial bonding is strong and reliable.

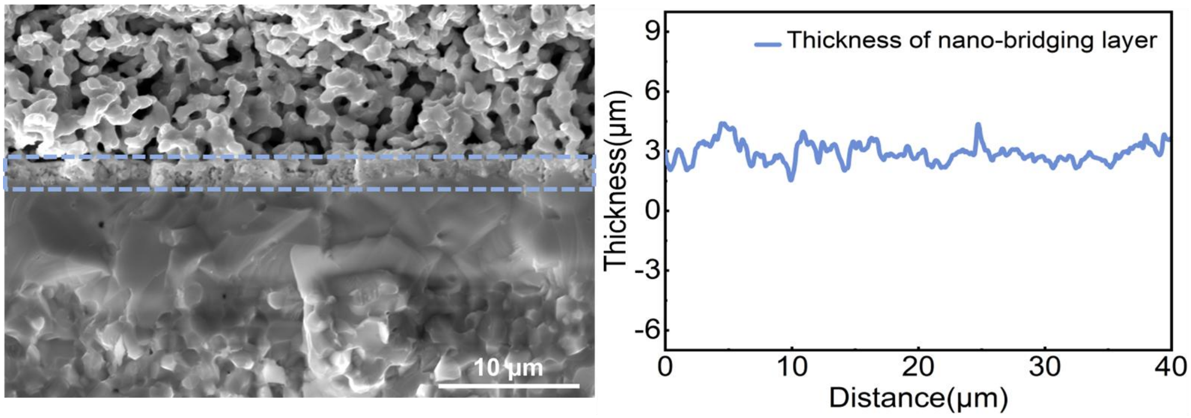


**Figure S4.** Boundaries of the blue-boxed ROI in the cross-sectional SEM and the local thickness at evenly spaced positions.

Using the 10 µm scale bar for pixel calibration, we segmented the upper and lower boundaries of the blue-boxed ROI in the cross-sectional SEM and sampled the local thickness at evenly spaced positions along ~40 µm. The resulting thickness-vs-distance profile is shown in Figure S. The interfacial layer is uniformly ~3 µm thick with small fluctuations (typical variation within ±0.5 µm over the 40 µm line scan), demonstrating good thickness uniformity.

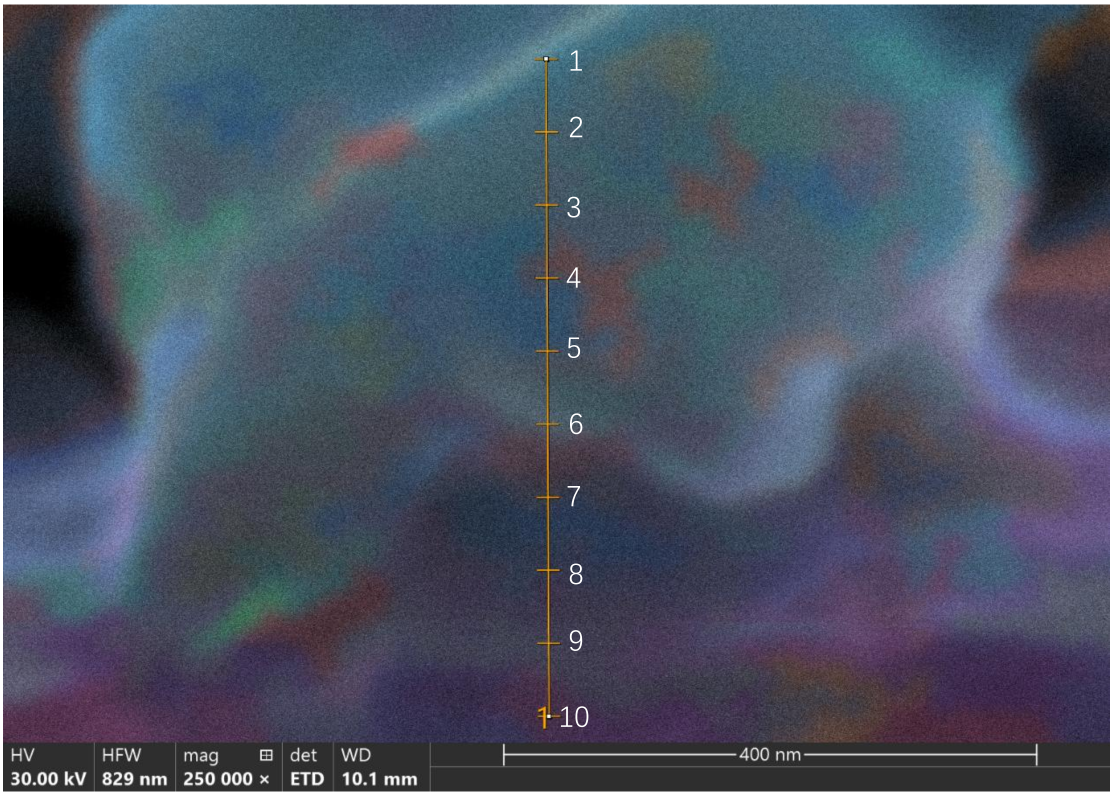


**Figure S5**. Line Scan Path Across the PNC73/BCZYYb Interface for Elemental Distribution Analysis.

EDS line scan results along the path shown in Figure S3, revealing the atomic percentage of key elements (Co, Ni, Y, Zr, Ba, Ce, Pr, and Yb) across the PNC/BCZYYb interface. A clear compositional transition is observed near 250–300 nm, indicating the boundary between the two phases. A continuous cation gradient smooths chemical-potential and defect-chemical discontinuities at the junction, thereby lowering the driving force for parasitic phase formation (e.g., Ba–Pr or Co–Zr oxides) and shrinking space charge layers associated with aliovalent dopants. Concomitantly, the gradual transition mitigates lattice/TEC mismatch and concentrates less stress during thermal cycling, promoting stronger interfacial bonding and crack suppression.

Mechanistically, the graded interphase acts as a mixed-transport region: protonic carriers (hydrated defects from BCZYYb) percolate into the Ni/Co-containing, electronically conductive PNC matrix, while PNC oxygen exchange sites persist toward the electrolyte. This extends the WOR/ORR active zone from a nominally sharp plane to a finite thickness (hundreds of nanometers), increases effective TPB density, and diminishes charge transfer barriers, manifesting as reduced interfacial polarization and faster electrode kinetics.

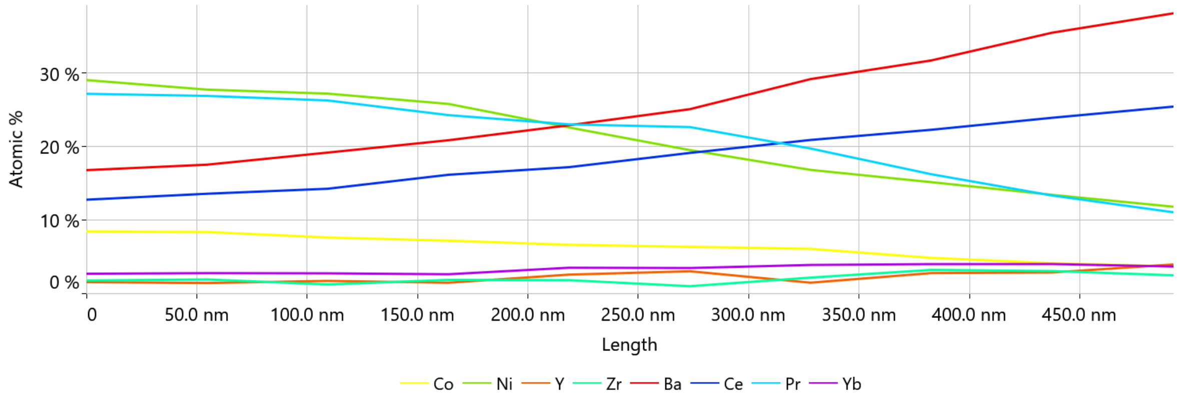


**Figure S6**. EDS Line Scan Showing Elemental Distribution Across the PNC–BCZYYb Interface.

The profile indicates a gradual decrease in Pr and Co concentrations and a corresponding increase in Ba and Zr content, confirming the transition from the PNC side to the BCZYYb side. The interface appears compositionally graded rather than abrupt, suggesting elemental interdiffusion across the boundary.

**Line 1**

Total Number of Counts: 10 640 412
Total Acquisition Time: 290 seconds
Average Count Rate: 36 691 cps
Acceleration Voltage: 30 kV

**Table S2**. Quantitative EDS Results of the PNC Phase from the Line Scan Region

| Element | Atomic % | Atomic % Error | Weight % | Weight % Error |
|---|---|---|---|---|
| Co | 6.3 | 0.0 | 3.2 | 0.0 |
| Ni | 20.8 | 0.0 | 10.5 | 0.0 |
| Y | 2.6 | 0.0 | 2.0 | 0.0 |
| Zr | 2.5 | 0.0 | 2.0 | 0.0 |
| Ba | 25.2 | 0.0 | 29.9 | 0.1 |
| Ce | 18.3 | 0.1 | 22.1 | 0.1 |
| Pr | 21.0 | 0.1 | 25.5 | 0.1 |
| Yb | 3.3 | 0.0 | 4.9 | 0.0 |

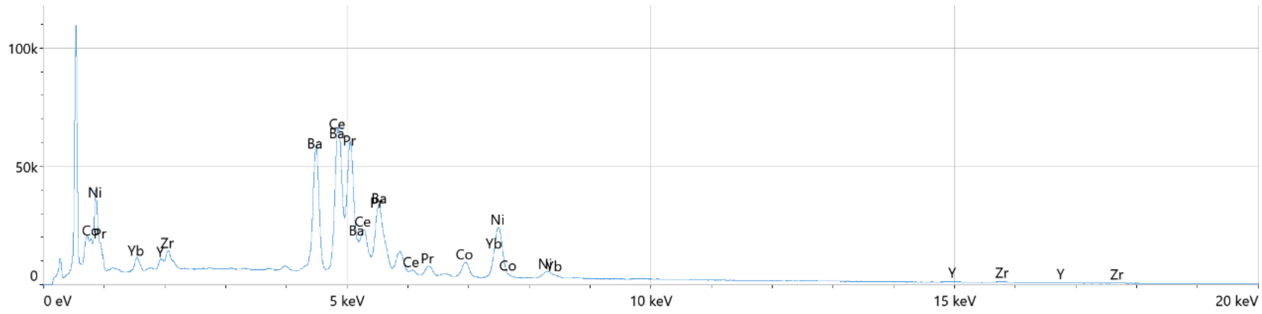


**Figure S7.** EDS Spectrum of the PNC Phase Confirming Elemental Composition.

**Line Point 1**

Total Number of Counts: 1 087 973
Total Acquisition Time: 29 seconds
Average Count Rate: 37 516 cps
Acceleration Voltage: 30 kV

**Table S3**. Quantitative EDS Results of the PNC Phase from the Starting Point Scan Region

| Element | Atomic % | Atomic % Error | Weight % | Weight % Error |
|---|---|---|---|---|
| Co | 8.4 | 0.1 | 4.6 | 0.1 |
| Ni | 29.0 | 0.1 | 15.7 | 0.0 |
| Y | 1.5 | 0.1 | 1.2 | 0.1 |
| Zr | 1.7 | 0.0 | 1.5 | 0.0 |
| Ba | 16.8 | 0.1 | 21.2 | 0.1 |
| Ce | 12.7 | 0.2 | 16.4 | 0.2 |
| Pr | 27.2 | 0.2 | 35.2 | 0.2 |
| Yb | 2.7 | 0.0 | 4.2 | 0.1 |

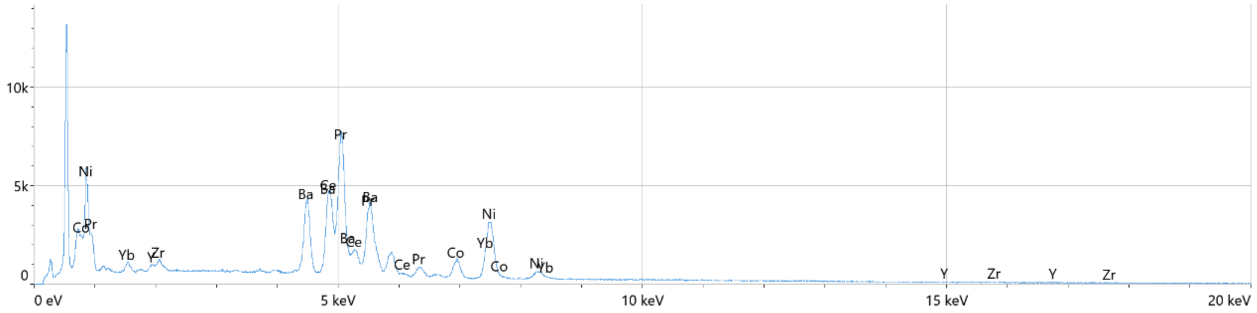


**Figure S8**. EDS Spectrum Acquired at the Starting Point of the Line Scan on the PNC Side.

**Line Point 2**

Total Number of Counts: 1 066 151
Total Acquisition Time: 29 seconds
Average Count Rate: 36 764 cps
Acceleration Voltage: 30 kV

**Table S4**. Quantitative EDS Results of the PNC Phase from Point 2 Scan Region

| Element | Atomic % | Atomic % Error | Weight % | Weight % Error |
|---|---|---|---|---|
| Co | 8.3 | 0.1 | 4.5 | 0.1 |
| Ni | 27.7 | 0.1 | 14.8 | 0.0 |
| Y | 1.4 | 0.1 | 1.1 | 0.1 |
| Zr | 1.9 | 0.0 | 1.6 | 0.0 |
| Ba | 17.5 | 0.1 | 21.9 | 0.1 |
| Ce | 13.5 | 0.1 | 17.3 | 0.1 |
| Pr | 26.9 | 0.2 | 34.5 | 0.2 |
| Yb | 2.7 | 0.0 | 4.3 | 0.1 |

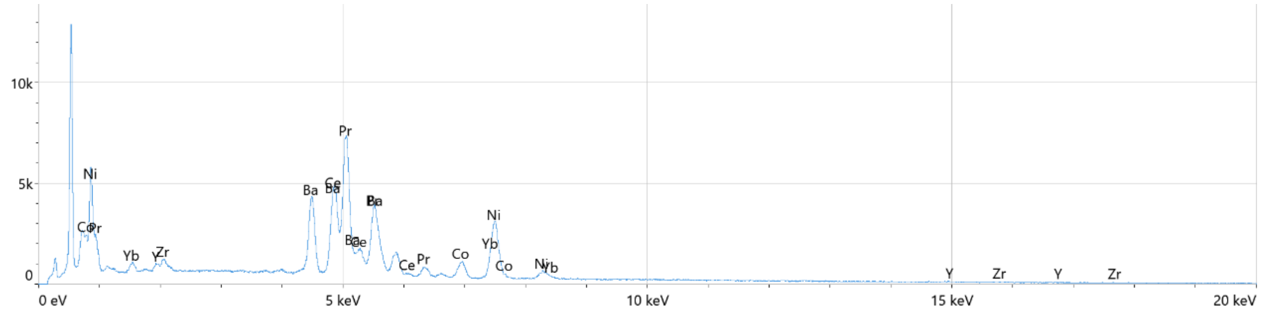


**Figure S9**. EDS Spectrum at Point 2: The PNC/BCZYYb Interface.

**Line Point 3**

Total Number of Counts: 1 053 311
Total Acquisition Time: 29 seconds
Average Count Rate: 36 321 cps
Acceleration Voltage: 30 kV

**Table S5**. Quantitative EDS Results of the PNC Phase from Point 3 Scan Region

| Element | Atomic % | Atomic % Error | Weight % | Weight % Error |
|---|---|---|---|---|
| Co | 7.6 | 0.1 | 4.0 | 0.1 |
| Ni | 27.2 | 0.1 | 14.4 | 0.0 |
| Y | 1.7 | 0.1 | 1.3 | 0.1 |
| Zr | 1.2 | 0.0 | 1.0 | 0.0 |
| Ba | 19.2 | 0.1 | 23.7 | 0.1 |
| Ce | 14.2 | 0.2 | 18.0 | 0.2 |
| Pr | 26.3 | 0.2 | 33.3 | 0.2 |
| Yb | 2.7 | 0.0 | 4.2 | 0.1 |

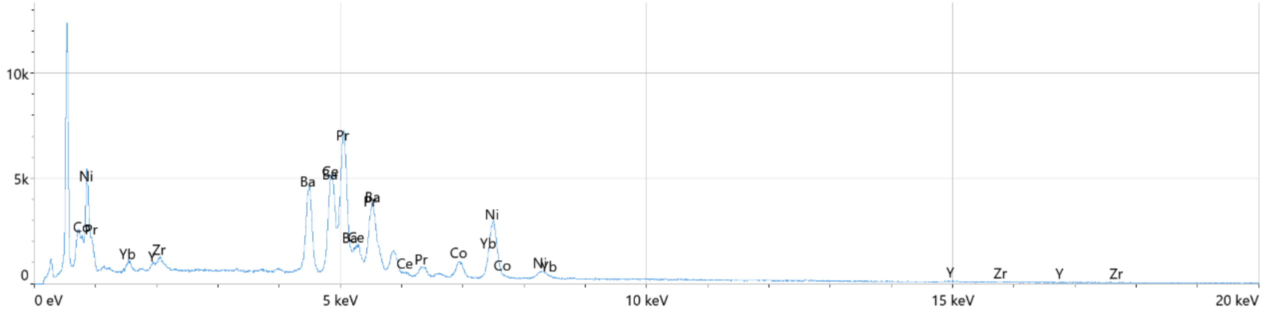


**Figure S10**. EDS Spectrum at Point 3: The PNC/BCZYYb Interface.

**Line Point 4**

Total Number of Counts: 1 048 319
Total Acquisition Time: 29 seconds
Average Count Rate: 36 149 cps
Acceleration Voltage: 30 kV

**Table S6**. Quantitative EDS Results of the PNC Phase from Point 4 Scan Region

| Element | Atomic % | Atomic % Error | Weight % | Weight % Error |
|---|---|---|---|---|
| Co | 7.2 | 0.1 | 3.8 | 0.1 |
| Ni | 25.8 | 0.1 | 13.5 | 0.0 |
| Y | 1.4 | 0.1 | 1.1 | 0.0 |
| Zr | 1.8 | 0.0 | 1.5 | 0.0 |
| Ba | 20.8 | 0.1 | 25.5 | 0.1 |
| Ce | 16.1 | 0.1 | 20.2 | 0.1 |
| Pr | 24.3 | 0.2 | 30.5 | 0.2 |
| Yb | 2.6 | 0.0 | 4.0 | 0.1 |

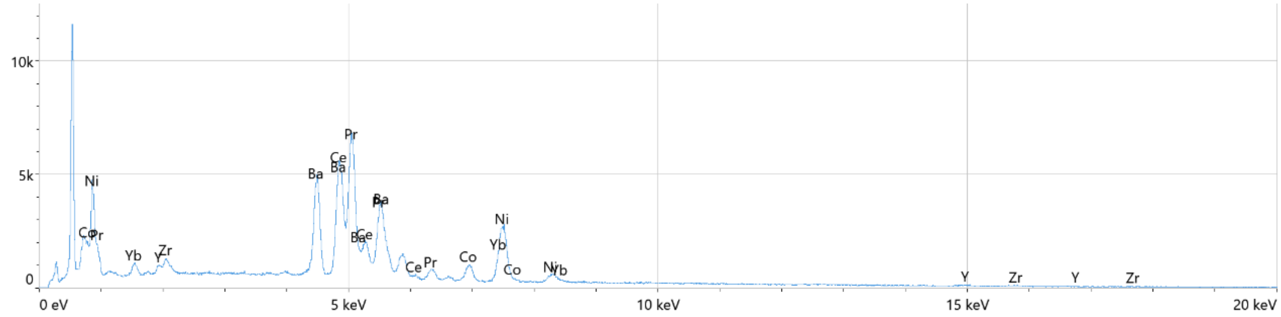


**Figure S11**. EDS Spectrum at Point 4: The PNC/BCZYYb Interface.

## Line Point 5

Total Number of Counts: 1 076 694
Total Acquisition Time: 29 seconds
Average Count Rate: 37 127 cps
Acceleration Voltage: 30 kV

**Table S7**. Quantitative EDS Results of the PNC Phase from Point 5 Scan Region

| Element | Atomic % | Atomic % Error | Weight % | Weight % Error |
|---|---|---|---|---|
| Co | 6.6 | 0.1 | 3.4 | 0.1 |
| Ni | 22.6 | 0.1 | 11.5 | 0.0 |
| Y | 2.5 | 0.1 | 2.0 | 0.1 |
| Zr | 1.8 | 0.0 | 1.4 | 0.0 |
| Ba | 22.9 | 0.1 | 27.3 | 0.1 |
| Ce | 17.2 | 0.2 | 20.9 | 0.2 |
| Pr | 23.0 | 0.2 | 28.2 | 0.2 |
| Yb | 3.5 | 0.0 | 5.2 | 0.1 |

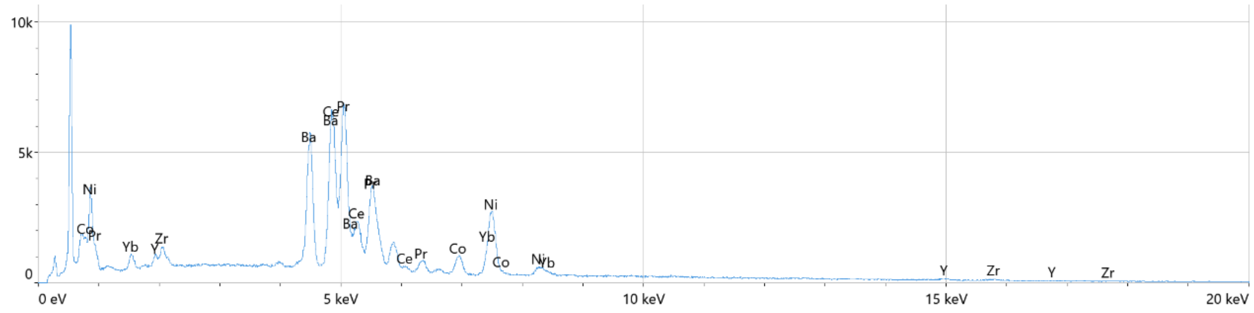


**Figure S12**. EDS Spectrum at Point 5: The PNC/BCZYYb Interface.

## Line Point 6

Total Number of Counts: 1 081 031
Total Acquisition Time: 29 seconds
Average Count Rate: 37 277 cps
Acceleration Voltage: 30 kV

**Table S8**. Quantitative EDS Results of the PNC Phase from Point 6 Scan Region

| Element | Atomic % | Atomic % Error | Weight % | Weight % Error |
|---|---|---|---|---|
| Co | 6.3 | 0.1 | 3.2 | 0.1 |
| Ni | 19.5 | 0.1 | 9.7 | 0.0 |
| Y | 3.0 | 0.2 | 2.3 | 0.1 |
| Zr | 1.0 | 0.0 | 0.7 | 0.0 |
| Ba | 25.1 | 0.1 | 29.2 | 0.1 |
| Ce | 19.1 | 0.2 | 22.7 | 0.2 |
| Pr | 22.6 | 0.2 | 27.1 | 0.2 |
| Yb | 3.4 | 0.0 | 5.0 | 0.1 |

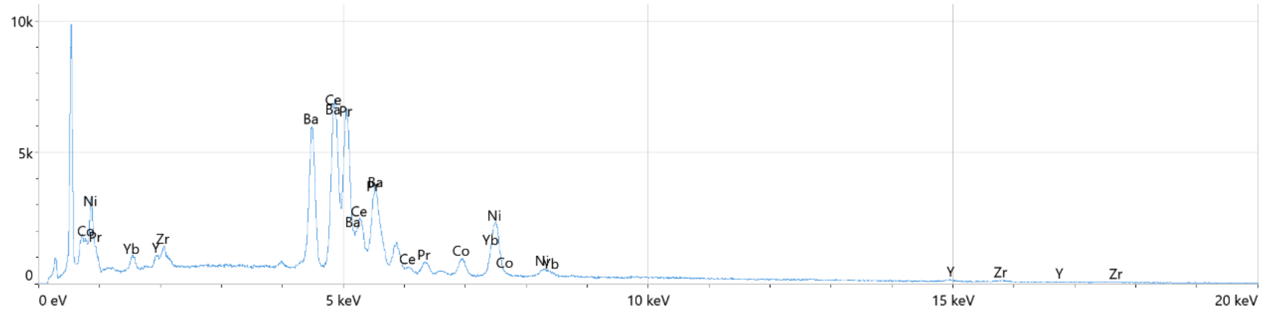


**Figure S13**. EDS Spectrum at Point 6: The PNC/BCZYYb Interface.

**Line Point 7**

Total Number of Counts: 1 059 472
Total Acquisition Time: 29 seconds
Average Count Rate: 36 534 cps
Acceleration Voltage: 30 kV
**Table S9**. Quantitative EDS Results of the PNC Phase from Point 7 Scan Region

| Element | Atomic % | Atomic % Error | Weight % | Weight % Error |
|---|---|---|---|---|
| Co | 6.1 | 0.1 | 3.0 | 0.1 |
| Ni | 16.8 | 0.1 | 8.2 | 0.0 |
| Y | 1.4 | 0.1 | 1.1 | 0.0 |
| Zr | 2.1 | 0.0 | 1.6 | 0.0 |
| Ba | 29.2 | 0.1 | 33.3 | 0.1 |
| Ce | 20.9 | 0.2 | 24.3 | 0.2 |
| Pr | 19.7 | 0.2 | 23.1 | 0.2 |
| Yb | 3.8 | 0.0 | 5.5 | 0.1 |

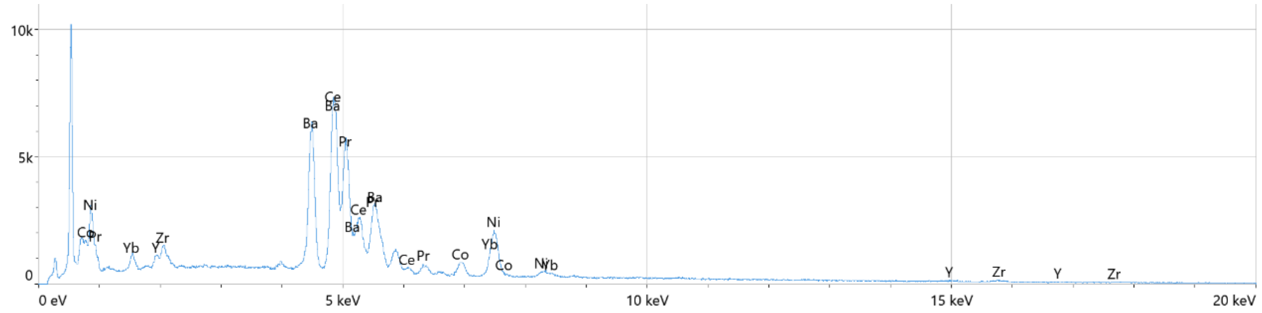


**Figure S14**. EDS Spectrum at Point 7: The PNC/BCZYYb Interface.

**Line Point 8**

Total Number of Counts: 1 055 889
Total Acquisition Time: 29 seconds
Average Count Rate: 36 410 cps
Acceleration Voltage: 30 kV

**Table S10**. Quantitative EDS Results of the PNC Phase from Point 8 Scan Region

| Element | Atomic % | Atomic % Error | Weight % | Weight % Error |
|---|---|---|---|---|
| Co | 4.8 | 0.1 | 2.3 | 0.1 |
| Ni | 15.1 | 0.1 | 7.3 | 0.0 |
| Y | 2.7 | 0.1 | 2.0 | 0.1 |
| Zr | 3.2 | 0.0 | 2.4 | 0.0 |
| Ba | 31.7 | 0.1 | 35.8 | 0.1 |
| Ce | 22.3 | 0.2 | 25.7 | 0.2 |
| Pr | 16.2 | 0.2 | 18.8 | 0.2 |
| Yb | 4.0 | 0.0 | 5.6 | 0.1 |

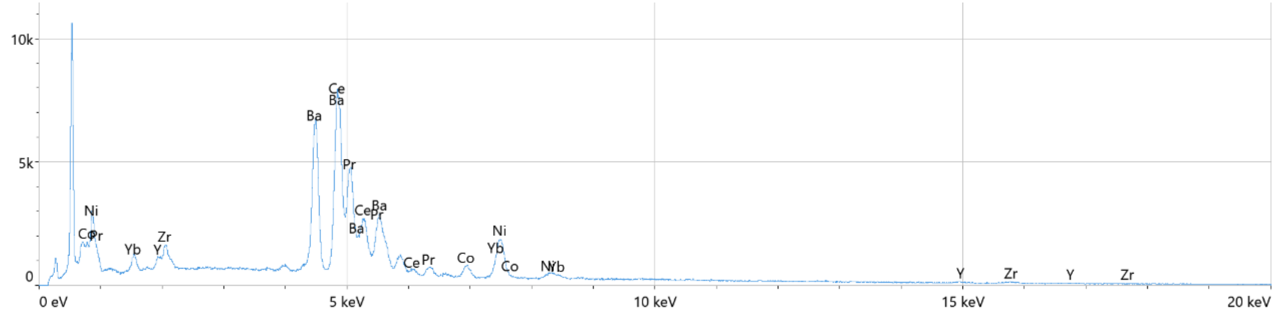


**Figure S15**. EDS Spectrum at Point 8: The PNC/BCZYYb Interface.

**Line Point 9**

Total Number of Counts: 1 054 056
Total Acquisition Time: 29 seconds
Average Count Rate: 36 347 cps
Acceleration Voltage: 30 kV

**Table S11**. Quantitative EDS Results of the PNC Phase from Point 9 Scan Region

| Element | Atomic % | Atomic % Error | Weight % | Weight % Error |
|---|---|---|---|---|
| Co | 4.1 | 0.1 | 2.0 | 0.1 |
| Ni | 13.4 | 0.1 | 6.4 | 0.0 |
| Y | 2.8 | 0.1 | 2.0 | 0.1 |
| Zr | 3.0 | 0.0 | 2.2 | 0.0 |
| Ba | 35.5 | 0.1 | 39.5 | 0.2 |
| Ce | 23.9 | 0.2 | 27.1 | 0.2 |
| Pr | 13.3 | 0.2 | 15.2 | 0.2 |
| Yb | 4.0 | 0.0 | 5.6 | 0.1 |

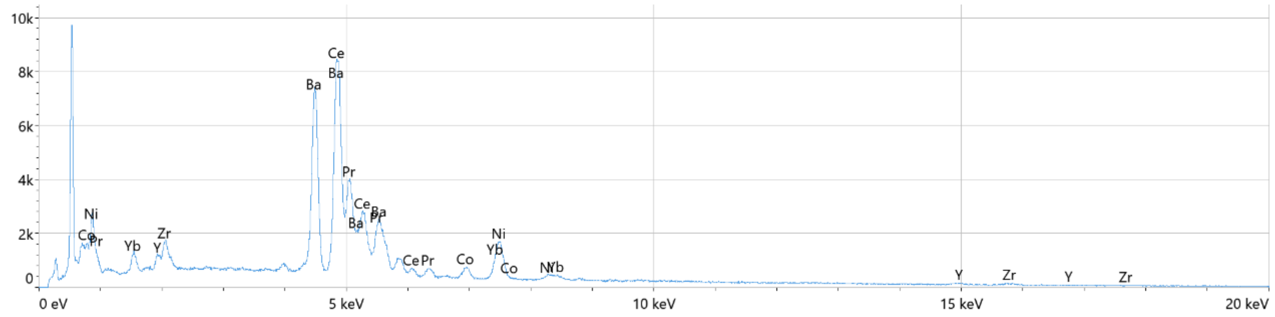


**Figure S16**. EDS Spectrum at Point 9: The PNC/BCZYYb Interface.

**Line Point 10**

Total Number of Counts: 1 057 516
Total Acquisition Time: 29 seconds
Average Count Rate: 36 466 cps
Acceleration Voltage: 30 kV

**Table S12**. Quantitative EDS Results of the PNC Phase from Point 10 Scan Region

| Element | Atomic % | Atomic % Error | Weight % | Weight % Error |
|---|---|---|---|---|
| Co | 3.7 | 0.1 | 1.7 | 0.1 |
| Ni | 11.8 | 0.1 | 5.6 | 0.0 |
| Y | 3.9 | 0.2 | 2.8 | 0.1 |
| Zr | 2.4 | 0.0 | 1.8 | 0.0 |
| Ba | 38.1 | 0.1 | 42.0 | 0.2 |
| Ce | 25.4 | 0.2 | 28.6 | 0.2 |
| Pr | 11.0 | 0.2 | 12.5 | 0.2 |
| Yb | 3.6 | 0.0 | 5.1 | 0.1 |

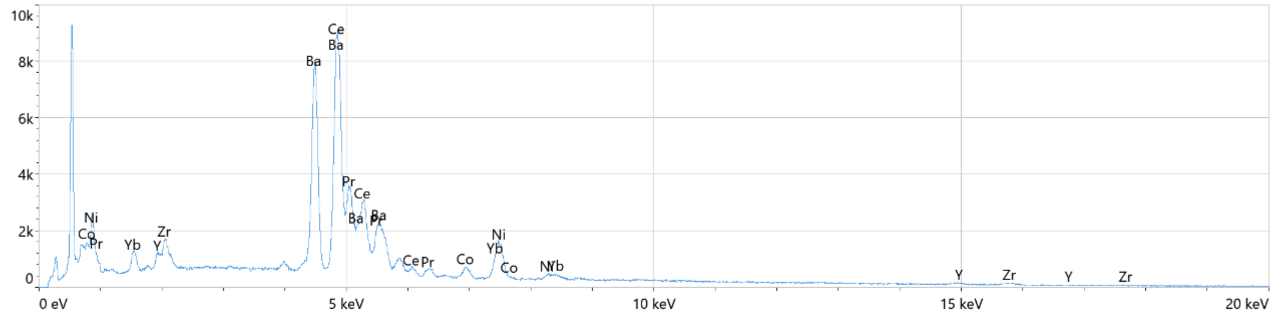


**Figure S17**. EDS Spectrum at Point 10: The PNC/BCZYYb Interface.

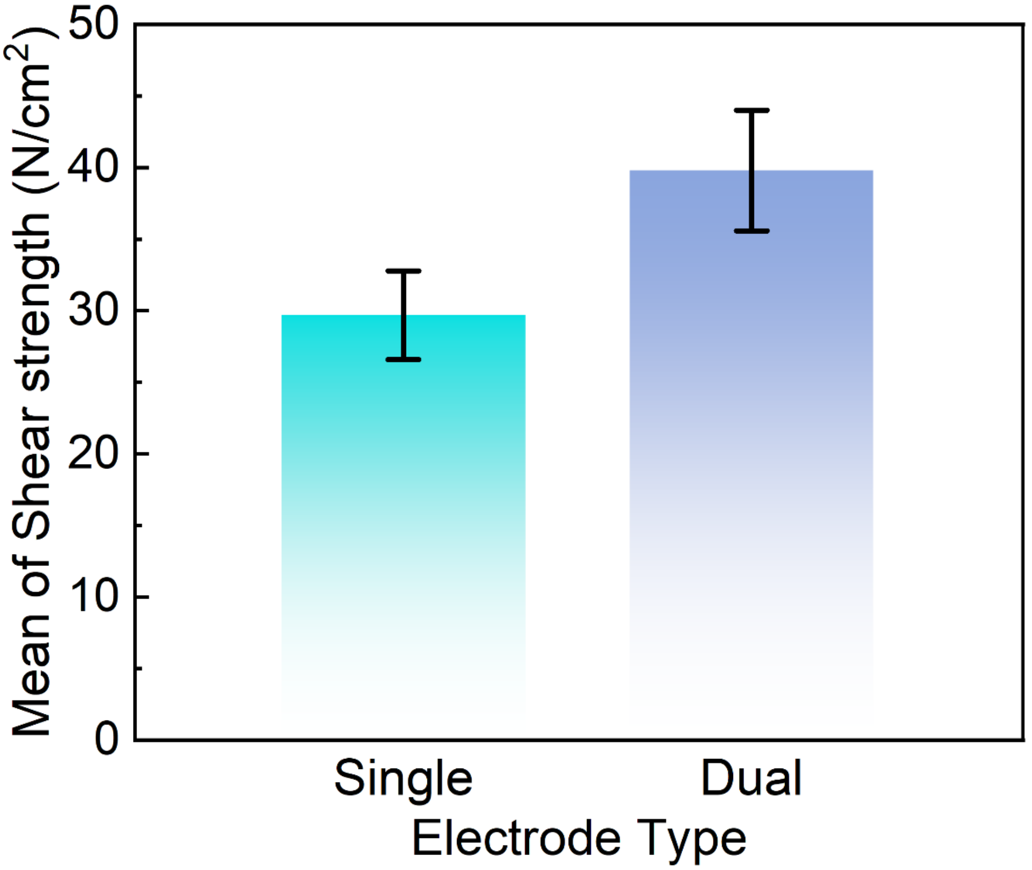


**Figure S18.** Error bar of shear strength (single-layer and dual-layer).

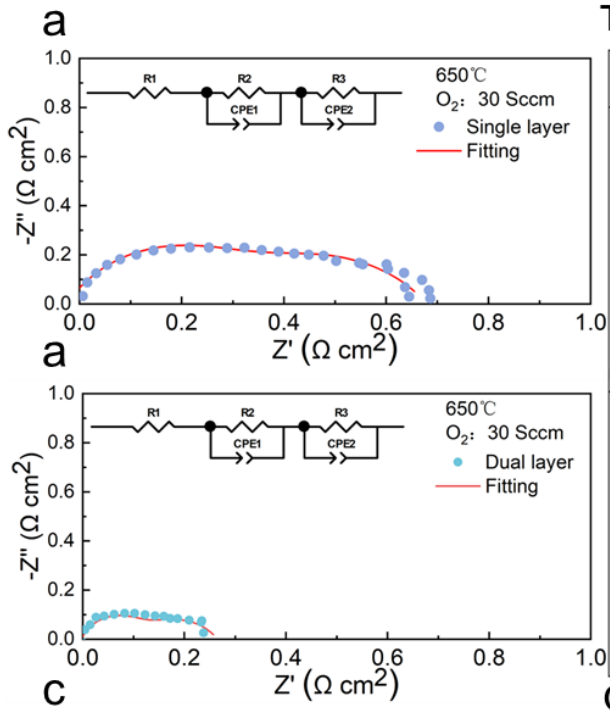


Table S13.Fitting Data

| Electrode Type | Element | Value | Error |
|---|---|---|---|
| Single Layer | R1 | 16.65 | 0.06515 |
| | R2 | 3.88 | 0.52823 |
| | CPE1-T | 0.0002480 | 9.0904E-05 |
| | CPE1-P | 0.65113 | 0.03757 |
| | R3 | 4.025 | 0.56227 |
| | CPE2-T | 0.0080342 | 0.0010563 |
| | CPE2-P | 0.55661 | 0.045473 |
| | Activation Energies | 0.779± 0.033e | |
| Dual Layer | R1 | 17.93 | 0.063698 |
| | R2 | 1.347 | 0.38532 |
| | CPE1-T | 0.0002401 | 0.00029724 |
| | CPE1-P | 0.79963 | 0.12725 |
| | R3 | 1.517 | 0.42072 |
| | CPE2-T | 0.016354 | 0.0075611 |
| | CPE2-P | 0.65681 | 0.14255 |
| | Activation Energies | 0.752± 0.043eV | |

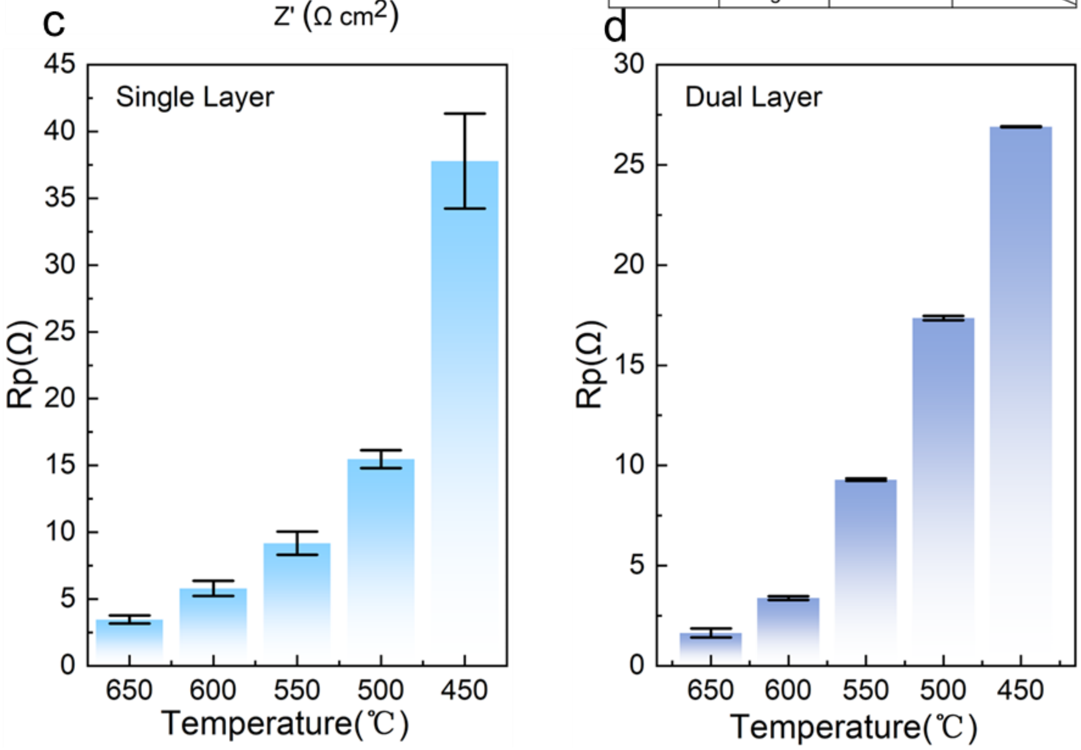


**Figure S19.** full equivalent-circuit fitting of all impedance spectra**. a** single-layer **b.** dual-layer. **c.** single-layer error bar from multiple cell tests. **d.** dual-layer error bar from multiple cell tests.

For both the single-layer and dual-layer samples, the impedance spectra are fit using the same equivalent circuit: a series resistance ($R_1$) in series with two parallel $R$-CPE (constant phase element) branches ($R_2$‖CPE1 and $R_3$‖CPE2). This model appropriately captures multiple interfacial, charge-transfer, and transport contributions to the total impedance.

In the single-layer sample, the Nyquist plot shows a large, depressed semicircle extending to ~0.6–0.7-ohm $cm^2$ on the real axis. This indicates substantial interfacial polarization. In contrast, the dual-layer sample shows a much smaller semicircle, terminating near ~0.2-ohm $cm^2$. The close match between the experimental data points and the fitted curve in both cases suggests that the equivalent circuit captures the dominant resistive processes.

Qualitatively, dual-layer architecture exhibits a significantly lower polarization response, implying improved interfacial charge transfer and better mechanical/electrochemical contact.

Reproducibility is presented in Figure S, Supporting Information as mean ± standard deviation (SD) from independently fabricated cells ($n = 3$ at each temperature). For each cell, multiple impedance scans were averaged, and uncertainties account for both fitting covariance and scan-to-scan variance.

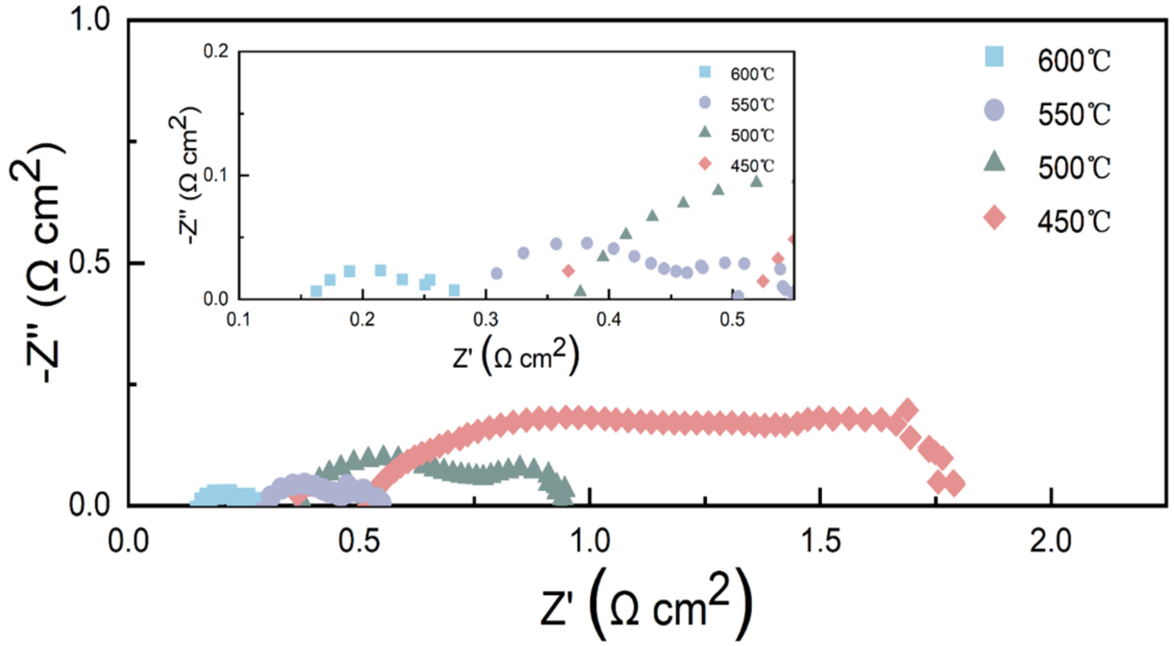


**Figure S20**. EIS spectra of Single-layer PNC73 in full cells measured under OCV conditions at 600 °C. Hydrogen (20 sccm) and oxygen (40 sccm) were respectively fed into the cells.

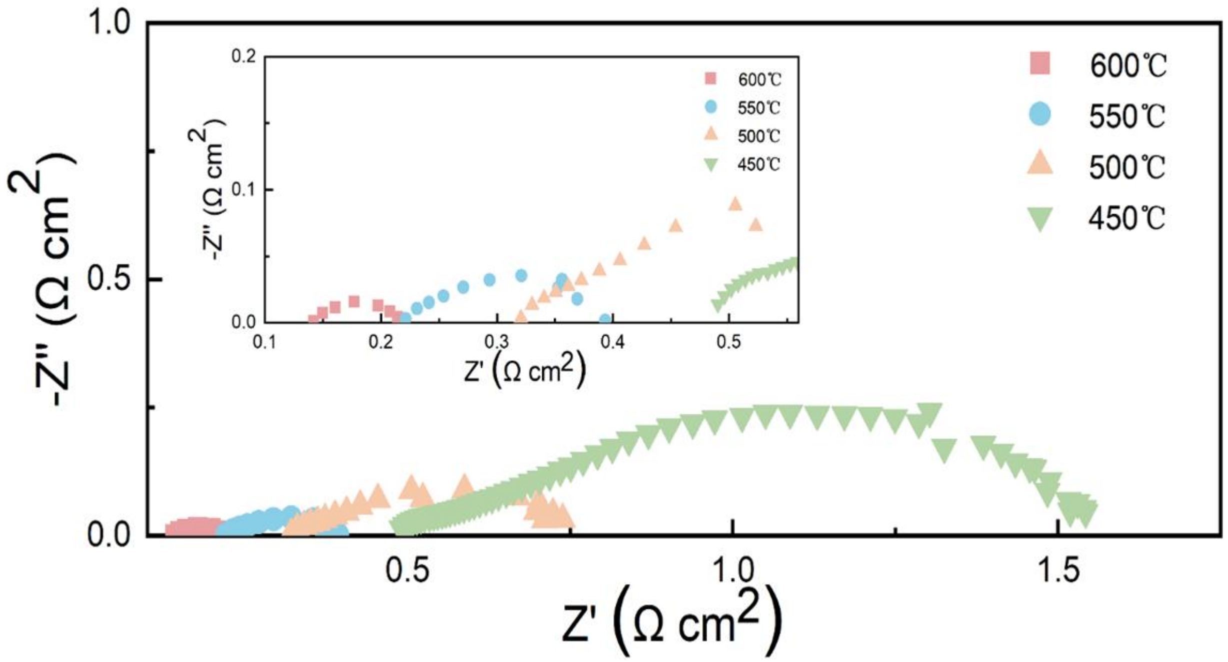


**Figure S21**. EIS spectra of Dual-layer PNC73 in full cells measured under OCV conditions at 600 °C. Hydrogen (20 sccm) and oxygen (40 sccm) were respectively fed into the cells.

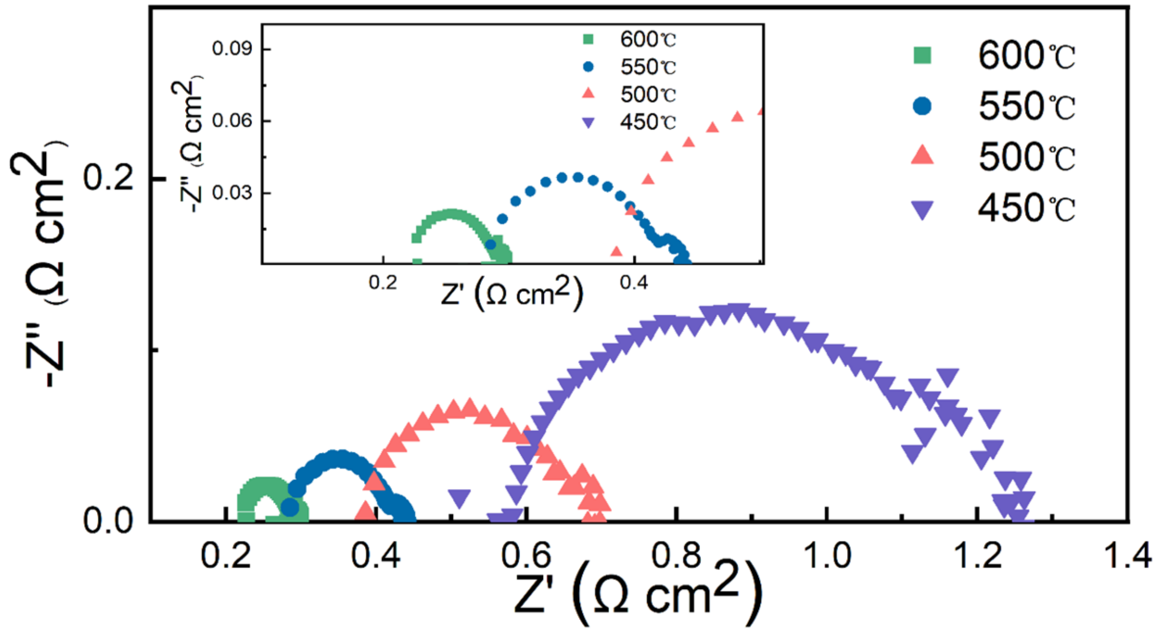


**Figure S22**. EIS spectra of Single-layer PNC73 measured under 1.30 V conditions at 600 °C. Hydrogen (20 sccm) and oxygen (40 sccm) were respectively fed into the cells.

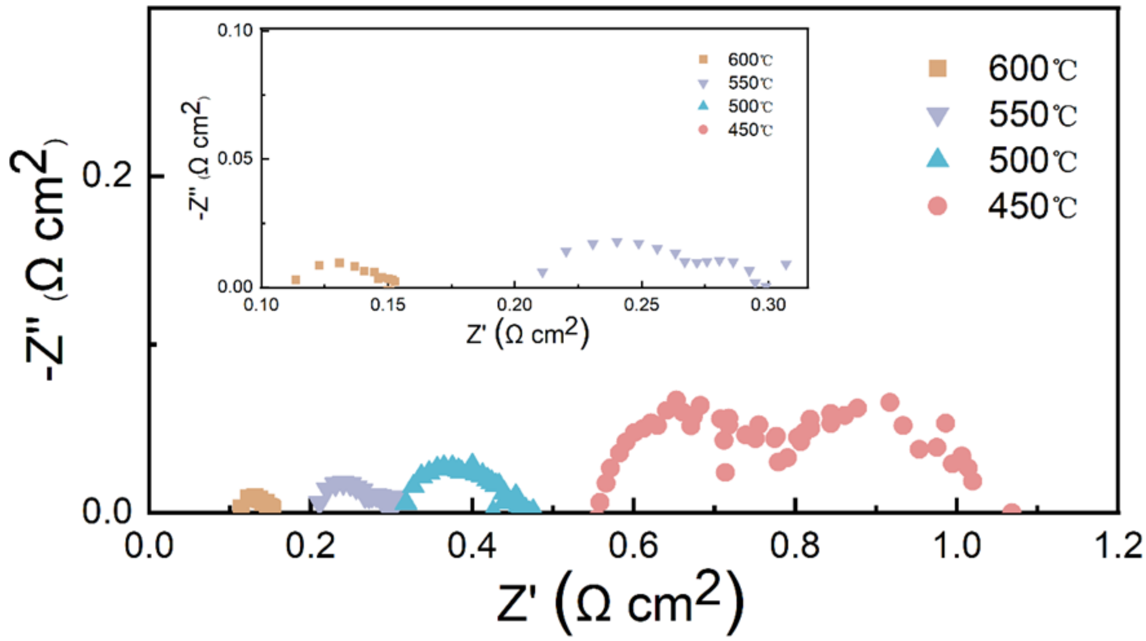


**Figure S23**. EIS spectra of Dual-layer PNC73 measured under 1.30 V conditions at 600 °C. Hydrogen (20 sccm) and oxygen (40 sccm) were respectively fed into the cells.

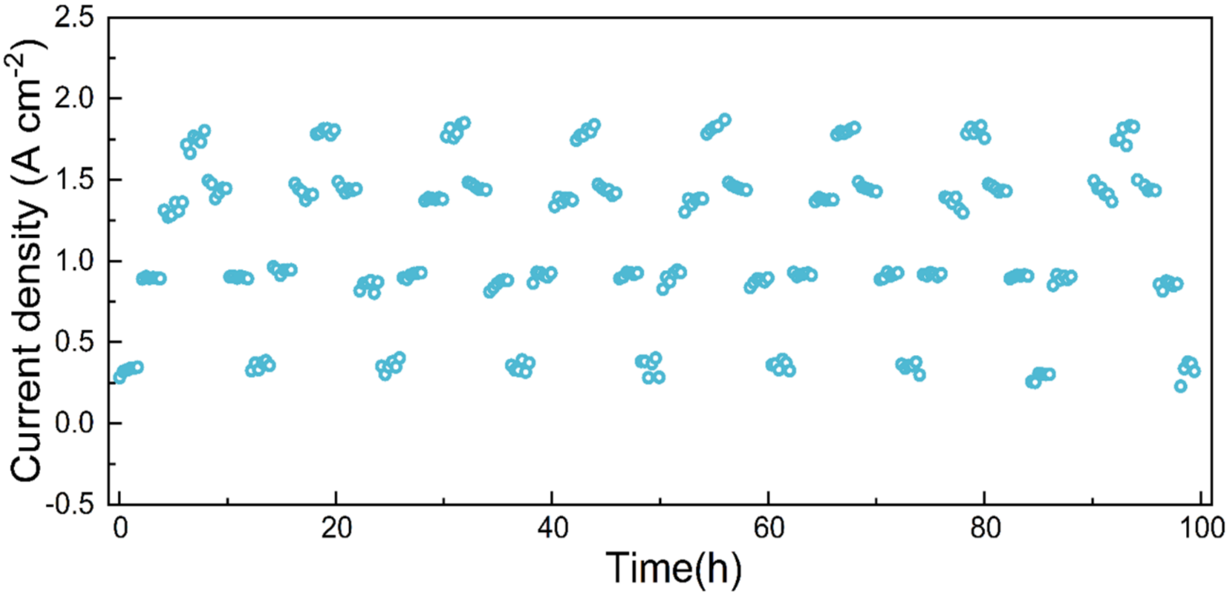


**Figure S24**. Reversible tests in fuel cell mode.

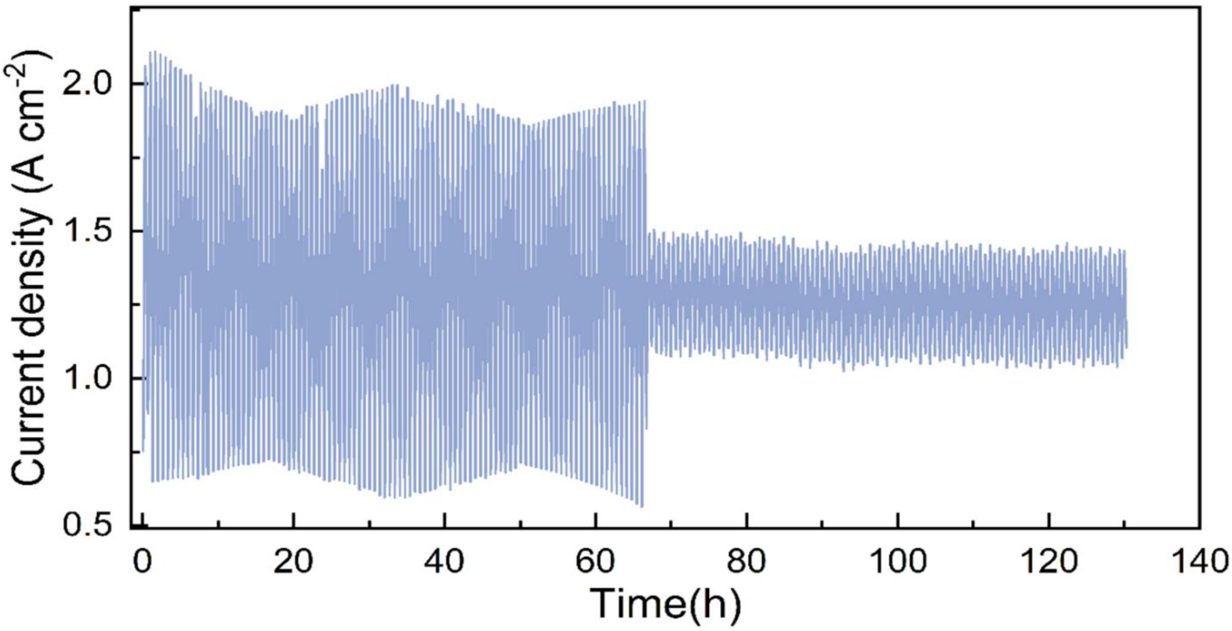


**Figure S25**. Transient tests (time-interval based) in fuel cell mode. All tests were conducted at 600 °C. Fuel cell mode was operated using H2 (20 sccm) for the anode and O2 (40 sccm) for the electrode.

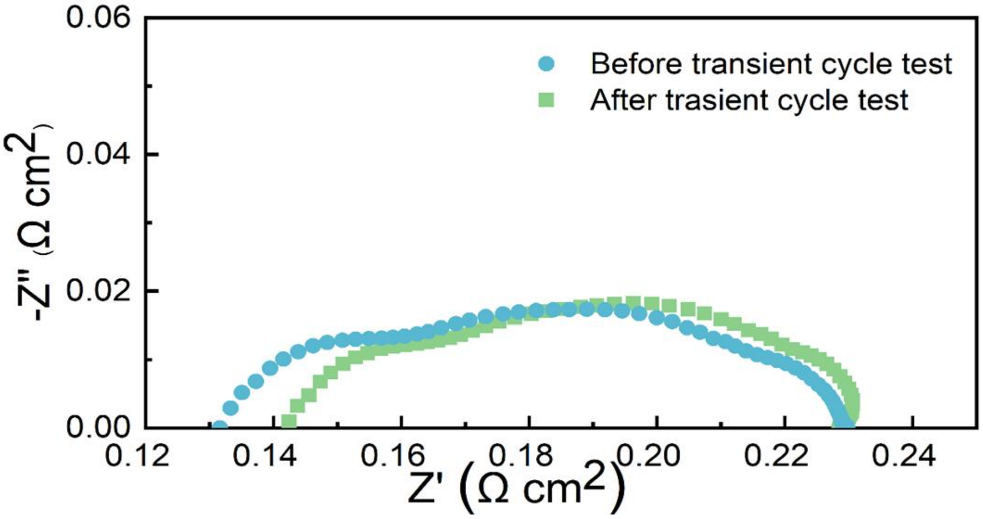


**Figure S26.** EIS Test before transient cycle and after transient cycle in fuel cell mode.

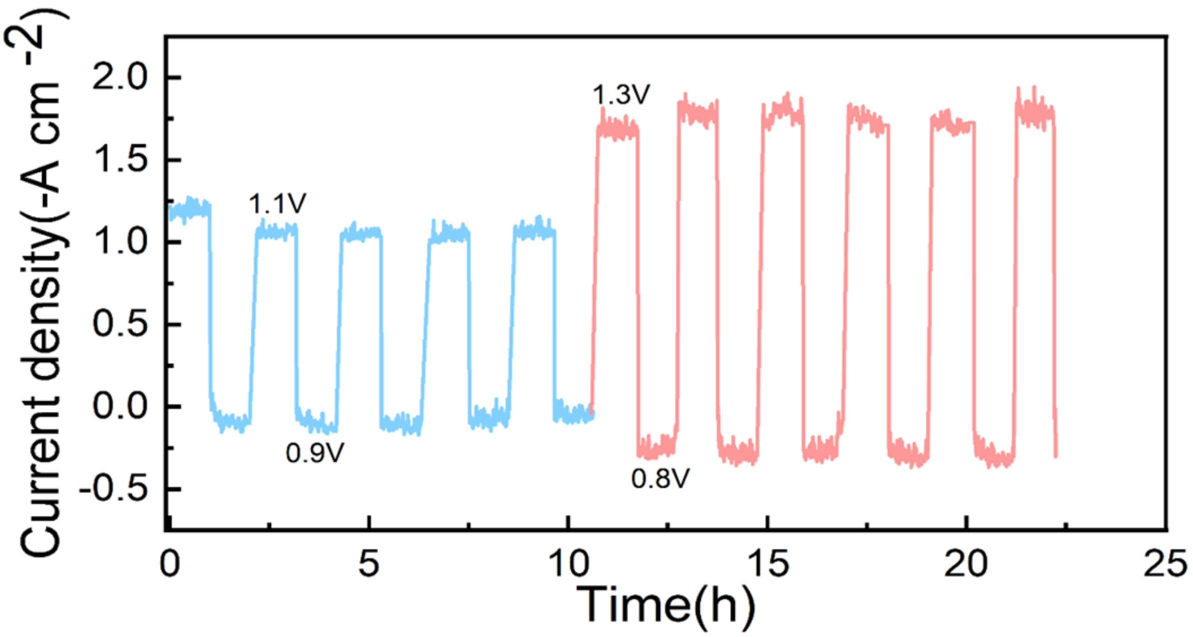


**Figure S27**. Reversible tests between 1.10V and 0.70 V, between 1.30V and 0.80 V for 21 hours (10 cycles).

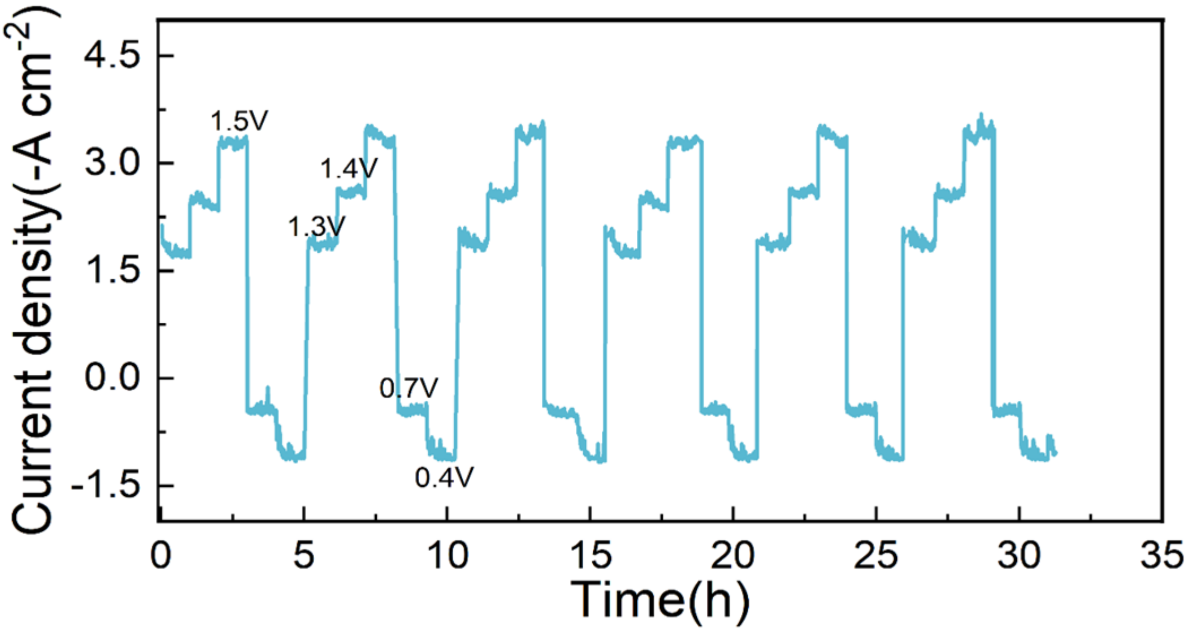


**Figure S28**. Reversible tests between electrolysis mode at 1.30 V, 1.40V, 1.50V and fuel cell mode at 0.70 V,0.40V for 21 hours (6 cycles).

**Hydrogen production and Faradaic efficiency calculations**

To assess electrolysis efficiency, a 20% or 40% steam–oxygen mixture was introduced to the HEN side, while 20 sccm of $H_2$ was supplied to the hydrogen electrode. The hydrogen output was quantified using a custom-designed flow meter, and Faradaic efficiency (FE) was calculated as:

$$FE(\%) = \frac{2F \cdot \mathcal{V}_{net}}{22400 \cdot \mathrm{I}} \times 100$$

Here, $\mathcal{V}_{net}$ represents the net flow rate ($\mathcal{V}_{outlet} - \mathcal{V}_{inlet}$) of $H_2$ generated in mL $s^{-1}$. $F$ is Faraday constant (96485 C $mol^{-1}$), and I is the applied current in amperes.

Energy efficiency (EE) for electrolysis was computed using:

$$EE(\%) = \frac{\Delta G^{\circ} \cdot FE(\%)}{2F \cdot \upsilon}$$

where $\upsilon$ is corresponding voltage (V) and $\Delta G^{\circ}$ is the Gibbs free energy at 600°C, 198500 J $mol^{-1}$.

**Table S14**. Faradaic efficiency test under different conditions.

| Air Conditions(atm) | Current Density (-A/cm²) | Faradaic efficiency |
|---|---|---|
| 0.2 | 0.6 | 80.66% |
| | 1.0 | 72.60% |
| | 1.4 | 63.38% |
| | 1.8 | 58.26% |
| | 2.2 | 47.66% |
| 0.4 | 0.6 | 87.38% |
| | 1.0 | 80.66% |
| | 1.4 | 74.90% |
| | 1.8 | 67.22% |
| | 2.0 | 58.66% |

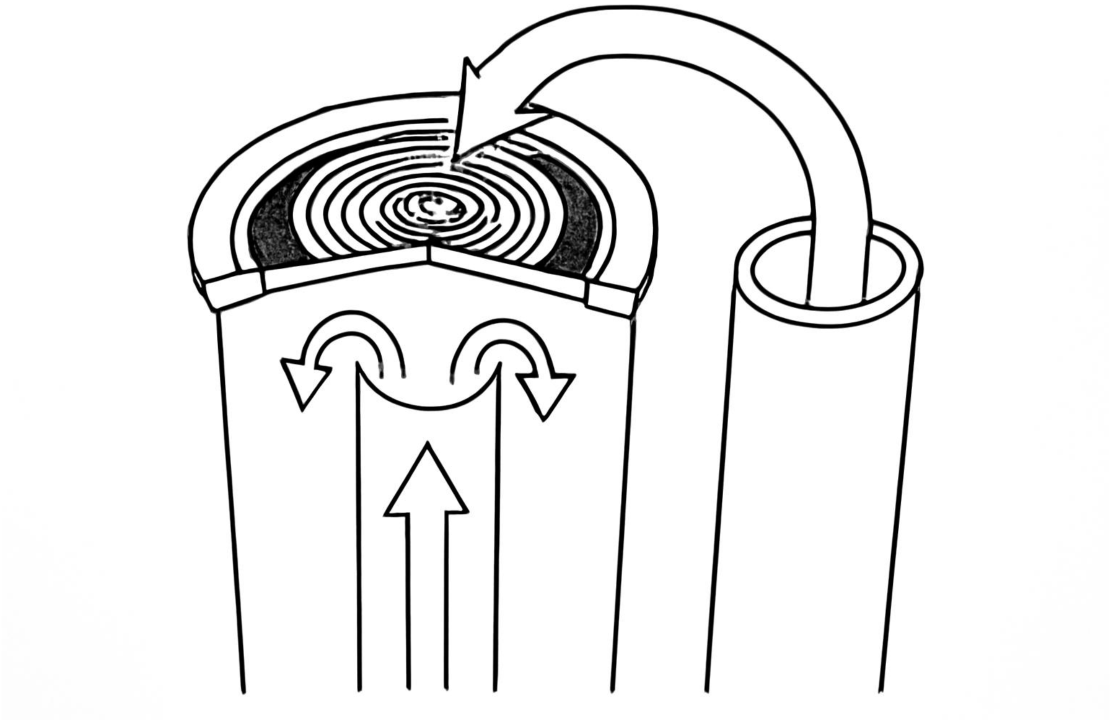

**Figure S29**. Cell test fixtures.

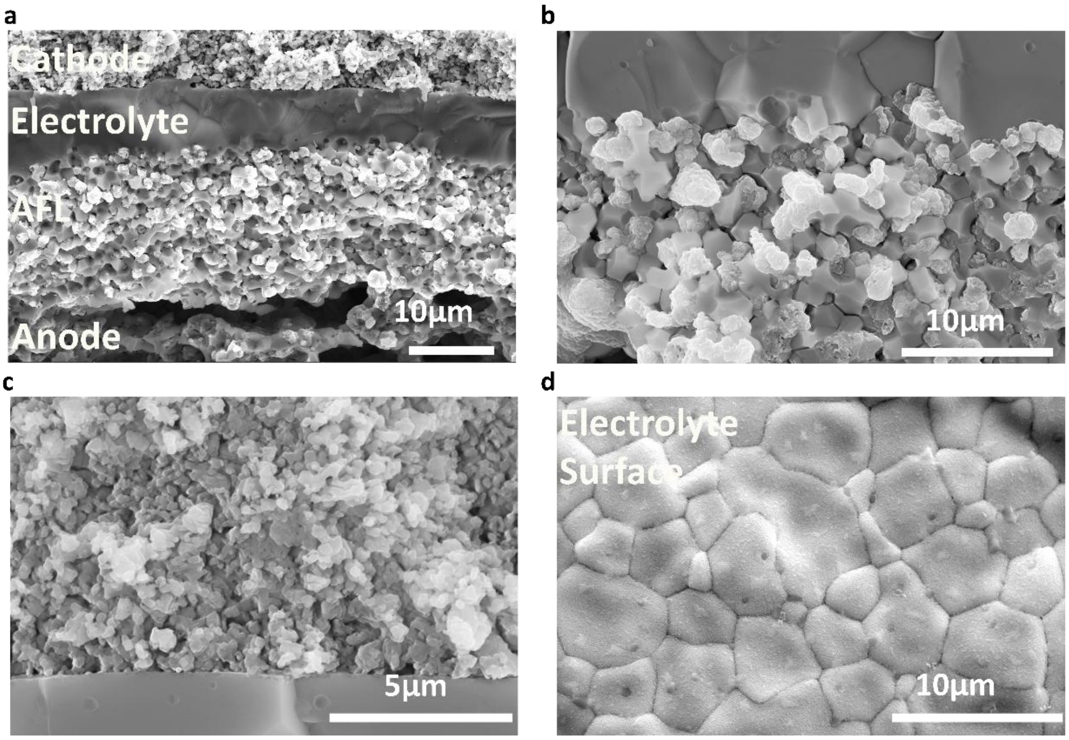


**Figure S30**. Microstructure examination for post-mortem cell with dual layer electrode. a. Cross-sectional view of the four layers in a single cell (oxygen electrode, electrolyte, AFL, and anode support layer). b. Electrolyte–AFL interface. c. PNC electrode–electrolyte interface. d. Electrolyte surface morphology.

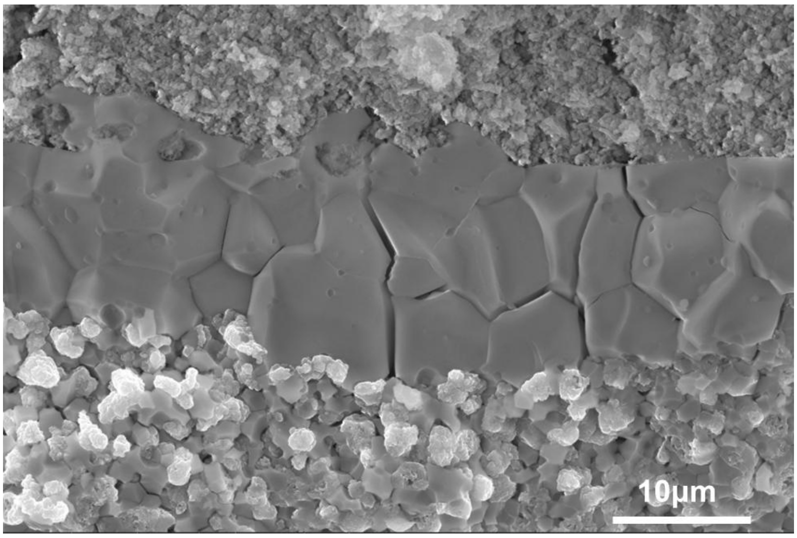


**Figure S31**. Microstructure examination for Cross-sectional view of after testing cell.

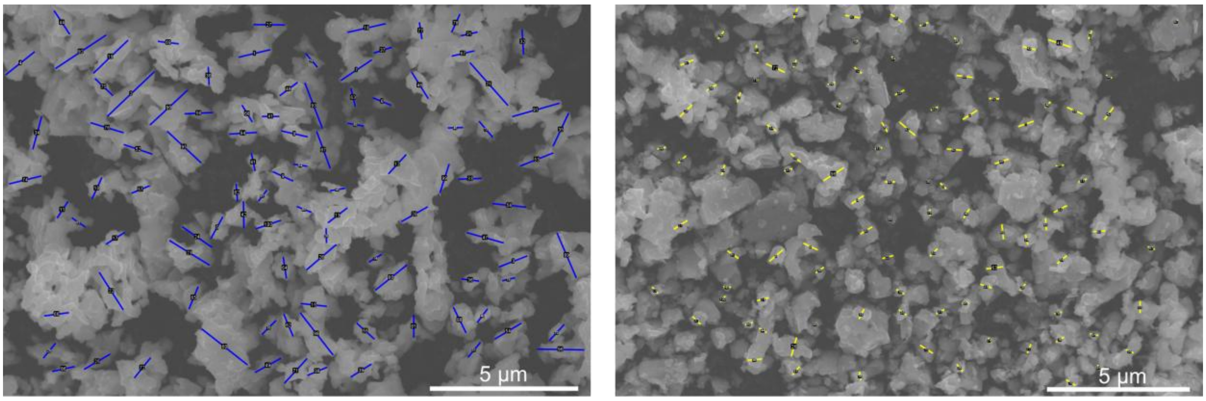


**Figure S32.** Measurement of pristine diameter and nano particles diameter.

**Table S15**. Statistical analysis of pristine diameter and nano particles diameter.

| Particle number | Pristine particles diameter (nm) | Nano particles diameter (nm) |
|---|---|---|
| 1 | 1098 | 529 |
| 2 | 2237 | 260 |
| 3 | 1031 | 557 |
| 4 | 1260 | 456 |
| 5 | 816 | 248 |
| 6 | 642 | 344 |
| 7 | 1267 | 311 |
| 8 | 915 | 564 |
| 9 | 744 | 506 |
| 10 | 786 | 350 |
| 11 | 645 | 260 |
| 12 | 880 | 278 |
| 13 | 819 | 191 |
| 14 | 491 | 340 |
| 15 | 870 | 239 |
| 16 | 669 | 292 |
| 17 | 735 | 278 |
| 18 | 1331 | 163 |
| 19 | 1644 | 266 |
| 20 | 1022 | 249 |
| 21 | 1972 | 376 |
| 22 | 1526 | 218 |
| 23 | 1620 | 350 |
| 24 | 1233 | 337 |

| | | |
|---|---|---|
| 25 | 1017 | 319 |
| 26 | 633 | 644 |
| 27 | 1054 | 516 |
| 28 | 1332 | 543 |
| 29 | 473 | 135 |
| 30 | 595 | 67 |
| 31 | 554 | 426 |
| 32 | 840 | 223 |
| 33 | 771 | 384 |
| 34 | 1147 | 317 |
| 35 | 1239 | 280 |
| 36 | 572 | 278 |
| 37 | 611 | 343 |
| 38 | 703 | 299 |
| 39 | 814 | 288 |
| 40 | 559 | 238 |
| 41 | 568 | 469 |
| 42 | 892 | 483 |
| 43 | 493 | 402 |
| 44 | 956 | 266 |
| 45 | 487 | 216 |
| 46 | 1069 | 460 |
| 47 | 1305 | 310 |
| 48 | 743 | 333 |
| 49 | 986 | 780 |
| 50 | 522 | 411 |
| 51 | 633 | 259 |
| 52 | 1013 | 470 |
| 53 | 1207 | 324 |
| 54 | 1054 | 383 |
| 55 | 804 | 377 |
| 56 | 1142 | 248 |
| 57 | 642 | 465 |
| 58 | 947 | 315 |
| 59 | 585 | 344 |
| 60 | 2011 | 334 |
| 61 | 594 | 426 |
| 62 | 806 | 460 |
| 63 | 626 | 543 |
| 64 | 724 | 143 |
| 65 | 818 | 94 |
| 66 | 831 | 543 |

| | | |
|---|---|---|
| 67 | 777 | 218 |
| 68 | 649 | 475 |
| 69 | 694 | 213 |
| 70 | 717 | 189 |
| 71 | 1138 | 135 |
| 72 | 865 | 681 |
| 73 | 690 | 257 |
| 74 | 1127 | 157 |
| 75 | 822 | 479 |
| 76 | 1171 | 259 |
| 77 | 547 | 290 |
| 78 | 653 | 344 |
| 79 | 416 | 206 |
| 80 | 1384 | 365 |
| 81 | 743 | 273 |
| 82 | 569 | 310 |
| 83 | 525 | 378 |
| 84 | 438 | 146 |
| 85 | 509 | 180 |
| 86 | 761 | 226 |
| 87 | 1338 | 435 |
| 88 | 967 | 530 |
| 89 | 1677 | 181 |
| 90 | 1750 | 109 |
| 91 | 1685 | 335 |
| 92 | 2106 | 678 |
| 93 | 1681 | 441 |
| 94 | 934 | 249 |
| 95 | 1587 | 231 |
| 96 | 1622 | 800 |
| 97 | 443 | 124 |
| 98 | 1849 | 202 |
| 99 | 1021 | 262 |
| 100 | 885 | 219 |
| **Average diameter** | **963.65** | **336.97** |
| **Average Radius** | **481.825** | **168.485** |

Using these radii, we calculated the surface energy per unit volume and correlated it with the observed microstructural and electrochemical trends. Smaller nanoparticles exhibit higher surface energy density, which enhances adhesion at the electrode interface and promotes

densification. This effect is consistent with the reduced interfacial impedance and improved polarization behavior observed in the dual-layer electrodes.

For a spherical nanoparticle, the per-particle surface energy scales with surface area,

$$E_{\mathrm{surf}} = \gamma\, 4\pi R^2 \qquad (2)$$

where $\gamma$is the interfacial energy and $R$ is the radius. Using the measured radius for the large and small populations ( $R_L = 481.825$ nm , $R_S = 168.485$ nm ), sintering kinetics and interfacial electrochemistry are governed by the surface-energy density of the ensemble. Since

$$V = \frac{4}{3}\pi R^3 \;\Rightarrow\; \frac{E_{\mathrm{surf}}}{V} = \frac{3\gamma}{R}, \qquad (3, 4)$$

the surface-energy density increases inversely with size.

$$\frac{(E/V)_S}{(E/V)_L} = \frac{R_L}{R_S} \approx 2.859, \quad (5)$$

In contrast, the small-particle mode ($R_S = 168.485$ nm) exhibits a 2.859× higher surface-energy density than the large-particle mode ($R_L = 481.825$ nm). This scaling rationalizes the observed trends (Fig. 7b): mixtures enriched in the nanoparticle mode exhibit faster sintering, larger bonded coverage at the interface, and consequently lower interfacial/polarization resistance.
